# Tumour-initiating cell-specific miR-1246 and miR-1290 expression converge to promote non-small cell lung cancer progression

**DOI:** 10.1038/ncomms11702

**Published:** 2016-06-21

**Authors:** Wen Cai Zhang, Tan Min Chin, Henry Yang, Min En Nga, Declan Patrick Lunny, Edwin Kok Hao Lim, Li Li Sun, Yin Huei Pang, Yi Ning Leow, Shanneen Rossellini Y Malusay, Priscilla Xin Hui Lim, Jeravan Zili Lee, Benedict Jian Wei Tan, Ng Shyh-Chang, Elaine Hsuen Lim, Wan Teck Lim, Daniel Shao Weng Tan, Eng Huat Tan, Bee Choo Tai, Ross Andrew Soo, Wai Leong Tam, Bing Lim

**Affiliations:** 1 Genome Institute of Singapore, Singapore 138672, Singapore; 2 Cancer Science Institute of Singapore, National University of Singapore, Singapore 117599, Singapore; 3 Department of Haematology–Oncology, National University Cancer Institute, Singapore 119074, Singapore; 4 Department of Pathology, National University Hospital, Singapore 119074, Singapore; 5 Institute of Medical Biology, Singapore 138648, Singapore; 6 Department of Respiratory Medicine, Tan Tock Seng Hospital, Singapore 308433, Singapore; 7 Department of Medical Oncology, National Cancer Centre Singapore, Singapore 169610, Singapore; 8 Saw Swee Hock School of Public Health, National University of Singapore, Singapore 117549, Singapore; 9 Department of Biochemistry, Yong Loo Lin School of Medicine, National University of Singapore, Singapore 117597, Singapore

## Abstract

The tumour-initiating cell (TIC) model accounts for phenotypic and functional heterogeneity among tumour cells. MicroRNAs (miRNAs) are regulatory molecules frequently aberrantly expressed in cancers, and may contribute towards tumour heterogeneity and TIC behaviour. More recent efforts have focused on miRNAs as diagnostic or therapeutic targets. Here, we identified the TIC-specific miRNAs, miR-1246 and miR-1290, as crucial drivers for tumour initiation and cancer progression in human non-small cell lung cancer. The loss of either miRNA impacted the tumour-initiating potential of TICs and their ability to metastasize. Longitudinal analyses of serum miR-1246 and miR-1290 levels across time correlate their circulating levels to the clinical response of lung cancer patients who were receiving ongoing anti-neoplastic therapies. Functionally, direct inhibition of either miRNA with locked nucleic acid administered systemically, can arrest the growth of established patient-derived xenograft tumours, thus indicating that these miRNAs are clinically useful as biomarkers for tracking disease progression and as therapeutic targets.

## Abstract (web summary)

miRNAs can function either as proto-oncogenes or tumour suppressors in several cancers; however their function in tumour initiating cells is unclear. Here, Zhang *et al*. show that tumour initiating cell-specific miR-1246 and miR-1290 promote lung cancer initiation and metastasis and could serve as prognostic markers.

## Graphical abstract

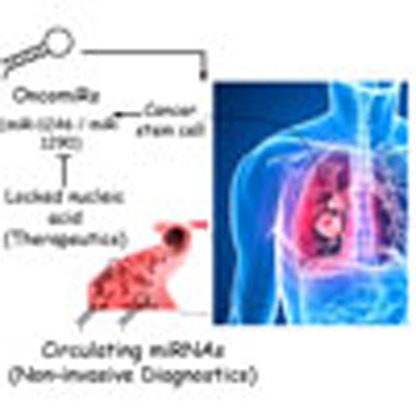

Lung cancer is the deadliest cancer worldwide, with non-small cell lung cancer (NSCLC) and small-cell lung cancer accounting for ∼85 and 15% of the incidences, respectively^1^. Despite advances in detection and improvements to standard of care, NSCLC is often diagnosed at an advanced stage and bears poor prognosis. Relapses are frequent after primary and adjuvant therapy, often evolving into a lethal metastatic disease^2^. These observations can, in part, be attributed to the highly heterogeneous nature of lung tumours that contain distinct tumoural and microenvironmental cell types, all of which contribute in varying degrees toward self-renewal, drug resistance, metastasis and relapse.

The tumour-initiating cell (TIC; also referred as cancer stem cell) model provides one explanation for the phenotypic and functional diversity among cancer cells in some tumours^3^. TICs have been demonstrated to be more resistant to conventional therapeutic interventions, and are key drivers of relapse and metastasis^4^,^5^,^6^. There is, therefore, increasing interests in developing strategies that can specifically target TICs with novel and emerging therapeutic modalities, thereby halting cancer progression and improving disease outcome^7^,^8^.

While significant efforts have focused on identifying agents and inhibitors that can disrupt the function of proteins, such as kinases and transcription regulators, necessary for TIC function, another avenue involves understanding the contribution of non-protein-coding molecules, and how they may be exploited as diagnostic and therapeutic targets^9^,^10^. MicroRNAs (miRNAs) represent a class of therapeutic targets that have been shown extensively to drive or inhibit cancer progression, and in some instances, may also be utilized as non-invasive biomarkers^11^,^12^,^13^,^14^. These findings, in part, resulted in the first miRNA mimic to enter Phase I clinic trials in patients with advanced hepatocellular carcinoma^15^.

MiRNAs have been found to function as either proto-oncogenes or tumour suppressors in almost all cancers through their dysregulated expression^16^. For instance, miR-17∼92 cluster has been widely documented to promote tumour formation in a variety of cancers^17^, whereas let-7 has shown conserved function as a tumour suppressor in lung and other cancers^18^,^19^. A few studies have begun to demonstrate the contribution of miRNAs in TICs either using cultured human cell lines or mouse models^20^,^21^,^22^,^23^, but these do not necessarily recapitulate their *bona fide* function in human tumours which tend to be more heterogeneous, and for which TICs can be better defined. Thus, there is a need to adopt the use of patient-derived tumour models and direct interrogation of patient materials for assessing the contributions of miRNAs and their diagnostic value in cancer.

Certain miRNAs have been detected as circulating cell-free molecules in the serum or plasma of cancer patients, and they appear to be useful as diagnostic or prognostic biomarkers for disease progression^24^,^25^,^26^. However, real-time tracking of circulating miRNAs level within individual patients across different time points, and how the levels impact their clinical response to ongoing therapy, has not been demonstrated, to the best of our knowledge.

In this study, we focused on dissecting the role of TIC-specific miRNAs that are found enriched in primary human lung tumours and their contribution towards disease progression and therapy response. We utilized lung TICs that were directly isolated from primary tumours and patient-derived tumourspheres to first identify and functionalize the previously poorly characterized miRNAs, miR-1246 and miR-1290, and demonstrated their crucial roles in tumour initiation and metastasis. We were able to track the circulating levels of both miRNAs in patients during the course of treatment to understand their response to ongoing therapy. Furthermore, we demonstrated the impact on tumour growth following the ablation of these miRNAs with locked nucleic acid (LNA) inhibitors, thus underscoring anti-miRNA strategies to be a viable therapeutic modality, at least, for NSCLCs.

## Results

### Identification of miRNAs restricted to TICs in NSCLC

**Figure 1:**
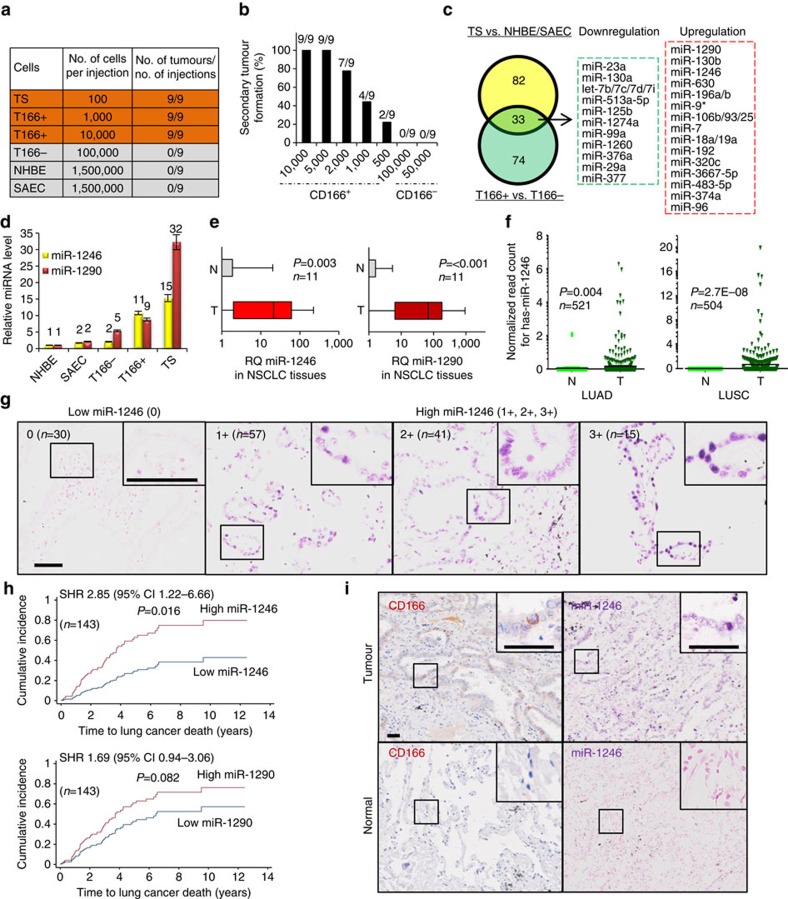
Heterogeneous expression of miR-1246 and miR-1290 in human NSCLC. (**a**) Tumour-formation efficiency of patient-derived tumour cell populations in NSG mice. T166^+^ or T166^−^, patient-derived CD166^+^ or CD166^−^ sorted tumour cells. T166^+^, T166^−^ and TS were generated from three patients. (**b**) Limiting dilution analysis of secondary tumour initiation by CD166^+^ and CD166^−^ cells from primary xenograft tumours. Number of xenografted tumours formed and number of cells injected are shown. *n*=3 patient samples. (**c**) Intersection of miRNAs enriched in lung TICs (TS and T166^+^) compared with non-TICs (NHBE, SAEC and T166^−^) by miRNA microarray. Two panels with downregulated and upregulated miRNAs lists are shown. *n*=3. (**d**) qRT–PCR analysis of both miR-1246 and miR-1290 in non-tumorigenic cells (NHBE, SAEC and T166^−^) and TICs (T166^+^ and TS). *n*=3. (**e**) qRT–PCR analysis of miR-1246 and miR-1290 levels by box plot in paired tumour and normal tissues in NSCLC. The median values for miR-1246 and miR-1290 levels in normal tissues were normalized as 1; *n*=11. Differences between groups were analysed using unpaired *t*-tests. (**f**) Expression levels of miR-1246 in normal (N) and tumour (T) tissues across human lung adenocarcinoma (LUAD) and lung squamous carcinoma (LUSC) using TCGA miRNA-Seq data. Differences between groups were analysed using unpaired *t*-tests. *n*=521 (LUAD), *n*=504 (LUSC). (**g**) ISH for miR-1246 in primary lung adenocarcinoma. The staining intensity was classified as low/absent (0) and high (1+, 2+ and 3+). Both the staining intensity and number of tumours are shown. Scale bar, 50 μm. (**h**) Cumulative incidence estimates on the effect of miR-1246 and miR-1290 expression, as determined by ISH, on NSCLC mortality. The effect estimate was quantified in terms of the subdistribution hazard ratio and its associated 95% confidence interval (CI). The *P* values were calculated using log-rank tests; *n*=143. (**i**) Staining CD166 (immunohistochemistry, left) and miR-1246 (ISH, right) on serial sections of tumour and normal lung tissues. Scale bar, 50 μm. Data are represented as the mean±s.e.m. RQ, relative quantification; TS, patient-derived tumourspheres.

To uncover miRNAs which are major regulators of lung TICs, we took advantage of our previous findings that patient-derived tumourspheres and CD166^+^ lung primary tumour cells are enriched for TICs^27^. Consistent with earlier data, we confirmed that patient-derived tumourspheres and CD166^+^ tumour cells were tumorigenic even when transplanted subcutaneously into mice at low cell numbers, whereas CD166^−^ tumour cells and two normal human primary lung epithelial cell lines (NHBE, human bronchial epithelial cells; and small airway epithelial cells (SAEC)) were completely devoid of this ability (Fig. 1a). To exclude non-tumour cells, we sorted for cells that were EPCAM^+^ carcinoma cells (Supplementary Fig. 1a). Limiting dilution cell transplantation analysis for tumour initiation showed that as few as 500 CD166^+^ cells could be serially propagated in immunodeficient *NOD.Cg-Prkdcscid Il2rgtm1Wjl/SzJ* (NSG) mice, whereas 100,000 CD166^−^ cells had no ability to form tumours in serial transplantation assays performed subcutaneously (Fig. 1b). These data confirmed both tumourspheres and CD166^+^ tumour cells to be enriched for TICs. To profile the miRNA expression from lung TICs and their differentiated progenies, which are non-tumour initiating, miRNA microarray was performed (Supplementary Fig. 1b). We first identified miRNAs which were enriched in CD166^+^ TICs relative to CD166^−^ non-TICs (*n*=3), and intersected these with miRNAs enriched in patient-derived tumourspheres relative to normal NHBE and SAEC. This method, utilizing two distinct manners of purifying for TICs, enabled us to robustly identify a conserved set of miRNAs which were exclusive to TICs but not non-TICs (Fig. 1c and Supplementary Table 1).

The top downregulated lung TIC-associated miRNAs include miR-23a, miR-130a, let-7 family, miR-513a-5p, miR-125b and miR-29a, whereas the top upregulated miRNAs include miR-1290, miR-130b, miR-1246, miR-630, miR-196a/b, miR-9/9* and miR-17∼92 cluster and its miR-106b∼25 analogues. Consistent with other studies, reduced let-7 miRNA family expression, which is associated with significantly shorter cancer patient survival, was found in TICs^18^,^28^. Similarly, miR-23a and miR-130a were shown to be downregulated in chronic myeloid leukaemia^29^, and miR-29a/b/c was frequently reduced in a variety of cancers that include lung cancer^30^. Conversely, upregulation of miR-17∼92 cluster and its paralogues miR-106b∼25, which were elevated in lung TICs, was found in several other cancers^31^, as these miRNAs promoted the rapid proliferation and undifferentiated phenotype of lung epithelial progenitor cells, as well as playing a role in embryonic lung development^32^,^33^. Other miRNAs that include miR-130b, miR-196a/b and miR-9/9*, similarly, were found to contribute towards the progression of other cancer types^34^,^35^,^36^.

From the compendium of candidate miRNAs, which were validated by quantitative RT–PCR (qRT–PCR; Supplementary Fig. 1c), at least several of these miRNAs have not been previously implicated in TIC function and oncogenesis. We chose to focus on miR-1246 and miR-1290 because they represent the topmost upregulated miRNAs among our TIC miRNA signature, and could be important drivers for cancer progression. Using another independent lung cancer patient cohort for which we generated tumourspheres, and also purified for CD166^+^ cells, we indeed confirmed miR-1246 and miR-1290 expression to be elevated more than fivefold in CD166^+^ TICs, and increased 6 and 30 times, respectively, in the corresponding patient-derived tumourspheres when compared with either normal lung epithelial cells or CD166^−^ non-TICs (Fig. 1d).

Because our initial evidence suggested that miR-1246 and miR-1290 could be restricted to lung TICs, we first sought to examine their expression patterns within human lung tumours as this serves to provide a clinically relevant context for studying their function. To broadly demonstrate the expression and specificity of certain miRNAs to NSCLC carcinoma cells, we analysed their levels in matched lung tumours and adjacent non-neoplastic tissues of the same individuals (*n*=11 pairs) by qRT–PCR using miR-16, miR-92 and miR-26b as endogenous controls that remained unaltered between tumour and normal tissues (Supplementary Fig. 1d). Both miR-1246 and miR-1290 showed consistent upregulation in tumours compared with their adjacent non-neoplastic tissues (*P*<0.01 for miR-1246, *P*<0.01 for miR-1290 by unpaired *t*-test; Fig. 1e). More importantly, the comparative expression of other miRNA candidates such as miR-130b, miR-23a and miR-125b, which were initially found to be also enriched in TICs, did not provide strong evidence that they were restricted to tumours, whereas miR-1246 and miR-1290 did (Supplementary Fig. 1e). Similarly, by analysing the miRNA sequencing-based expression of a far larger cohort of paired tumour and adjacent tissues deposited in The Cancer Genome Atlas (TCGA), we observed a highly significant elevation of miR-1246 in tumour samples, relative to adjacent tissues (Fig. 1f and Supplementary Table 2).

We next assessed whether the expression of miR-1246 and miR-1290 might be heterogeneous among the tumours of different NSCLC patients, and the implications for disease outcome. By *in situ*-hybridization (ISH) assay on tissue microarrays from a cohort of 143 patients (*n*=197 tumour cores; Fig. 1g), miR-1246 expression negatively correlated with patient survival (*P*=0.016 by log-rank test), while miR-1290 had a weaker correlation (*P*=0.082 by log-rank test; Fig. 1h and Supplementary Tables 3 and 4). Patients bearing tumours with higher miR-1246 expression, as assessed by staining intensity, showed elevated subdistribution hazard ratio compared with those harbouring tumours with lower miR-1246 expression (2.85, 95% confidence interval 1.22–6.66) (Fig. 1h). We further sought to verify the above findings with another cohort of lung cancer patients, this time analysing the miRNA-sequencing data set from TCGA. Consistently, higher miR-1246 or miR-1290 expression in tumours was associated with shorter patient survival periods (*P*=0.007 and *P*=0.024 by log-rank test, respectively; Supplementary Fig. 1f,g).

Since we initially found miR-1246 to be enriched in flow cytometry purified CD166^+^ cells, we proceeded to verify whether the miRNA was also found within TICs present in patient tumour sections. To compare the cellular expression of miR-1246 and CD166 protein, ISH and immunohistochemistry were first performed separately on serial sections of both malignant and normal lung tissues. Mir-1246 was strongly localized to the cytoplasm and nucleus in tumour cells, while remained weak or undetectable in most of the normal lung epithelial cells (Supplementary Fig. 1h). Similarly, CD166 positivity was predominately detected in the cytoplasm and cell membrane in tumour cells and almost absent in normal lung tissues (Fig. 1i and Supplementary Table 5). To directly correlate the expression pattern of miR-1246 with CD166, we combined ISH and immunohistochemistry on the same tissue or tumour section. MiR-1246 expression was predominantly restricted to CD166^+^ cells in primary NSCLC tumours, and largely absent in CD166^−^ tumour cells and normal lung epithelial cells (Supplementary Fig. 2a). *χ*^2^-Analysis in NSCLC tissues showed a strong correlation between the intensity of miR-1246 expression and miR-1290 expression by ISH (*P*<0.001 by Student's *t*-test; Supplementary Fig. 2b).

### MiR-1246 and miR-1290 confer tumorigenicity

**Figure 2:**
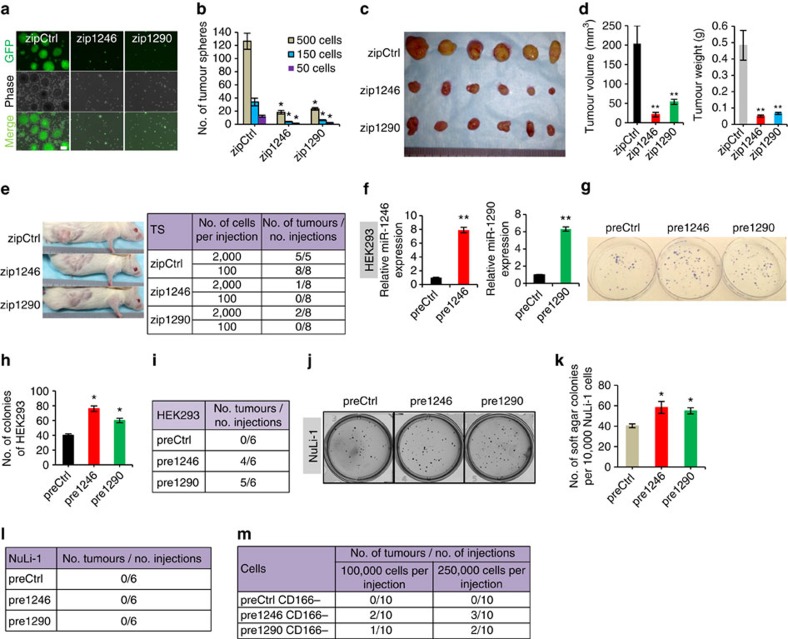
miR-1246 and miR-1290 contribute towards transformation and lung tumorigenesis. (**a**) Sphere-formation assay for tumoursphere cells treated with either miR-1246 knockdown (zip1246) or miR-1290 knockdown (zip1290). A total of 500 cells were seeded into each 10-cm dish. Spheres containing >50 cells were counted on day 13. Scale bar, 200 μm. (**b**) Limiting dilution analysis of sphere-formation efficiency for tumoursphere cells treated with either zip1246 or zip1290. 50, 150 and 500 cells were plated; *n*=3. (**c**,**d**) Images (**c**) and quantitative analysis of volume and mass (**d**) of tumours formed 28 days after subcutaneous transplantation of 100,000 tumoursphere cells that were treated with either zip1246 or zip1290; *n*=6. (**e**) Limiting dilution analysis of tumour initiation by tumoursphere cells treated with either zip1246 or zip1290. In all, 100 and 2,000 cells were transplanted subcutaneously and tumour formation was evaluated 90 days later. The representative mouse images on transplantation (left) and the number of xenografted tumours/number of injections are shown; *n*=8. (**f**) qRT–PCR analysis of miR-1246 and miR-1290 expressions in HEK293 infected with either pre-miR-1246 (pre1246) or pre-miR-1290 (pre1290) overexpression. (**g**,**h**) Colony-formation assay (**g**) and quantification (**h**) in adherent conditions of HEK293 treated with either pre1246 or pre1290 overexpression. In all, 100 cells were plated; *n*=3. (**i**) Xenograft tumour-formation efficiency of HEK293 treated with either pre1246 or pre1290. A total of 100,000 cells were transplanted subcutaneously and tumour formation was evaluated 60 days later; *n*=6. (**j**,**k**) Soft-agar colony-formation assay (**j**) and quantification (**k**) in NuLi-1 treated with either pre1246 or pre1290. A total of 10,000 cells were plated and the colonies were stained by INT after 28 days. (**l**) Xenograft tumour-formation efficiency of NuLi-1 treated with either pre1246 or pre1290. A total of 1,000,000 cells were transplanted subcutaneously and tumour formation was evaluated 60 days later; *n*=6. (**m**) Xenograft tumour formation of CD166^−^ tumour cells from xenograft tumour treated with pre1246 or pre1290. A total of 100,000 and 250,000 cells were transplanted subcutaneously and tumour formation was evaluated 90 days later; *n*=10. All error bars represent±s.e.m. and statistical significance was calculated using Student's *t*-test; *P<0.05, **P<0.01.

The highly enriched expression of miR-1246 and miR-1290 in lung CD166^+^ TICs, but not in CD166^−^ cells and normal lung epithelial cells, strongly suggests these two miRNAs to be crucial for tumour initiation and establishment. To test this, we utilized highly specific miRZip lentiviral anti-miR-1246 and anti-miR-1290 to knockdown miR-1246 and miR-1290 in lung tumourspheres and assessed their tumorigenic potential in cell cultures and in mice. Dissociated tumourspheres were initially plated on either soft agar or 2D adherent cell cultures—assays which select for the growth of bulk cancer cells, including differentiated cancer cell populations. Interestingly, this resulted only in a small decrease in colony numbers, indicating that the loss of miR-1246 and miR-1290 did not impact cell growth and proliferation under these cell culture conditions (Supplementary Fig. 3a–d). However, when tumourspheres bearing either miR-1246 or miR-1290 knockdown were subjected to extended periods of culture in serum-free sphere-forming condition that selects for TICs and stem cells, their numbers were markedly reduced by at least 5.4-fold (Fig. 2a). In a limiting dilution assay, sphere-formation efficiency was consistently reduced when 500, 150 and 50 cells were plated (Fig. 2b). When tumourspheres were transplanted into immune-compromised mice, those bearing knockdown of either miR-1246 or miR-1290 alone markedly inhibited tumour growth (Fig. 2c,d). To confirm that the loss of miR-1246 or miR-1290 impacted tumour initiation, we transplanted TS cells that were knocked down for either miRNA in limiting dilution cell numbers. While control-treated TS cells continued to form tumours with 100 cells, zip1246- or zip1290-treated cells were severely inhibited in their tumour initiation capability when 2,000 cells were transplanted, and were completely ablated of this ability with 100 cells, thus underscoring the role of these miRNAs in tumour initiation (Fig. 2e).

Because lung TICs and cancer progression were dependent on miR-1246 or miR-1290, we assessed whether these miRNAs could confer tumorigenic potential to otherwise normal-like or non-tumorigenic cells. Exogenous introduction of either miR-1246 or miR-1290 into immortalized human embryonic kidney cells (HEK293) modestly increased proliferation and colony formation *in vitro* (Fig. 2f–h and Supplementary Fig. 3e), but surprisingly, was able to confer on these otherwise non-tumorigenic cells and their tumorigenic potential, as gauged by the ability of the miRNA-bearing cells to form tumours efficiently (Fig. 2i). In all, 66.7% (4/6) and 88.3% (5/6) of mice bearing miR-1246- and miR-1290-overexpressing HEK293 cells, respectively, grew tumours, whereas none of the mice transplanted with control-treated cells formed tumours. To test the oncogenic potential of miRNAs in more physiologically relevant cell systems, we introduced either miR-1246 or miR-1290 into immortalized human lung epithelial cells (NuLi-1). The overexpression of either miRNAs increased soft-agar colony formation *in vitro*, but could not initiate tumours even when 1 × 10^6^ cells were xenografted into NSG mice (Fig. 2j–l). This indicates that the miRNAs could drive neoplastic transformation of normal-like lung cells *in vitro* but did not confer full tumorigenic potential. More strikingly, overexpression of either miR-1246 or miR-1290 in non-tumorigenic CD166^−^ cancer cells was able to confer tumorigenic ability, as demonstrated by the formation of tumours when 100,000 cells were transplanted (Fig. 2m).

### MiR-1246 and miR-1290 are required for lung cancer metastasis

**Figure 3:**
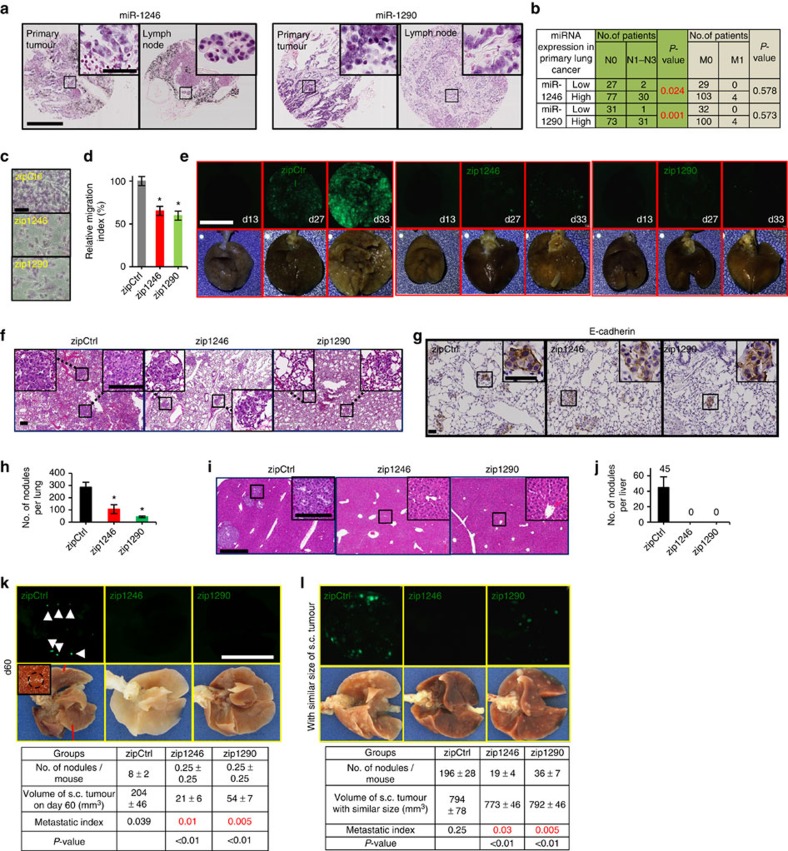
The metastatic ability of lung TICs is dependent on miR-1246 and miR-1290. (**a**) ISH for miR-1246 and miR-1290 in paired primary lung adenocarcinoma and lymph node metastases. Scale bars, 600 μm (50 μm in the inset). (**b**) Association between miR-1246 or miR-1290 expression in primary tumours by ISH and status of lymph node (N) and distant metastases (M). (**c**,**d**) Transwell migration assay and quantification of tumoursphere cells treated with either zip1246 or zip1290. The migrated cells were stained with Giemsa (**c**) and counted 6 h after plating with 100,000 cells (**d**); *n*=3. Scale bars, 50 μm. (**e**) Fluorescence and bright-field images of mouse lung for assessing metastasis. A total of 1 × 10^6^ tumoursphere cells treated with either zip1246 or zip1290 were injected through the tail-vein. Lung metastatic nodules on day 13, 27 and 33 after transplantation are shown. Scale bars, 1 cm. (**f**–**h**) H&E (**f**) and E-cadherin immunohistochemistry staining (**g**) for mouse lung sections as shown in **e** (day 27). Quantitative analysis of the number of metastatic pulmonary nodules per lung is shown (**h**). Scale bar, 200 μm (**f**) and 50 μm (**g**); *n*=4. (**i**,**j**) H&E staining for mouse liver sections after injecting 1 × 10^6^ tumoursphere cells on day 27 via tail vein (**i**). Quantitative analysis of the number of metastatic nodules in the liver per mouse is shown (**j**). Nodules (see inset) are shown in higher magnification. Scale bar, 700 μm (inset, 200 μm); *n*=4. (**k**,**l**) Fluorescence and bright-field images (top panel) of the mouse lung for assessing metastasis. A total of 1 × 10^6^ tumoursphere cells treated with either zip1246 or zip1290 were injected subcutaneously. Whole-lung tissues were collected on day 60 (**k**) and until the subcutaneous tumours reached a similar size (12 mm in diameter) (**l**) after transplantation. The metastatic nodules are shown in higher magnification (see inset). Quantitative analysis of the number of metastatic lung nodules is shown (lower panel). *n*=4. Scale bars, 1 cm. All error bars represent±s.e.m. and statistical significance was calculated using Student's *t*-test; *P<0.05.

More recent findings have indicated that subpopulations of carcinoma cells, which bear properties of TICs, help seed metastases at distant organs^37^,^38^. This led us to wonder whether miR-1246 and miR-1290 could confer metastatic traits to lung tumour cells. We first assessed the expression of these miRNAs in cancer cells that metastasized to either the lymph node or distant organs. To determine the correlation between miRNA expression levels in primary tumours and lymph node metastasis, we performed ISH for the miRNAs in paired primary lung tumours and corresponding lymph nodes (*n*=143 patients). Primary tumours that contain high miR-1246 or miR-1290 expression tended to correlate with the detection of metastatic tumour cells within lymph nodes, whereas those expressing low levels of the miRNAs did not (Fig. 3a,b). Hence, there was a strong association between expression of these miRNAs in primary tumour and lymph node metastasis (*P*=0.024 and *P*=0.001 by Student's *t*-test, respectively). Of note, however, we did not observe a strong correlation for the miRNA expression levels between primary tumours and incidence of distant metastases; this is likely attributed to the very few cases of paired tumour and distant metastases we were able to obtain. Overall, the results suggest that miR-1246 and miR-1290 could contribute towards the metastatic abilities of lung tumour cells.

To test the migratory and invasive roles of these miRNAs in tumourspheres, we first performed transmembrane migration and matrigel invasion assays for dissociated tumoursphere cells. Both the migration (Fig. 3c,d) and invasion capabilities (Supplementary Fig. 4a,b) were markedly impaired on knockdown of either miR-1246 (zip1246) or miR-1290 (zip1290), thus suggesting that the miRNAs were necessary for the invasion, at least *in vitro*. We further sought to examine whether miR-1246 and miR-1290 were indeed directly mediating the metastasis of lung TICs in animal models. We initially performed experimental metastasis analyses by directly introducing the same number of tumoursphere cells containing zip1246, zip1290 or zip-control, into the lungs of immune-compromised NSG mice through tail-vein injection; this allowed us to examine their extravasation and colonization abilities—key stages of the metastatic cascade. After 5 weeks, metastatic nodules in the lungs and liver were markedly reduced on knockdown of either miR-1246 or miR-1290 in tumoursphere cells before tail-vein injection (Fig. 3e–j and Supplementary Fig. 4c).

Subsequently, we transplanted tumoursphere cells expressing either zip1246 or zip1290 subcutaneously into NSG mice to determine their impact on spontaneous metastasis from primary tumours. Consistent with previous findings, transplanting large numbers of tumoursphere cells (1 × 10^6^), which contained either zip1246 or zip1290, gave rise to tumours that were reduced in size relative to control-treated cells. The latter cells seeded metastatic lung nodules efficiently 60 days post-transplantation, whereas miRNA-knockdown cells were deficient in this regard (Fig. 3k and Supplementary Fig. 4d). However, owing to differences in size of primary tumours arising from treated and control cells, it could be likely that larger tumours are more capable of disseminating metastatic cells. To account for this difference, we calculated the metastatic index, which normalizes the number of metastatic nodules to the size of primary tumours. The metastatic index was indeed four to eight times lower in mice containing tumoursphere cells expressing either zip1246 or zip1290, relative to control-treated cells (Fig. 3k). Alternatively, we allowed zip1246- and zip1290-expressing tumoursphere cells to form tumours, over a prolonged period of time, until they were approximately similar in size to control tumours (12 mm in diameter), and subsequent examined the number of metastatic lung nodules. The number of lung nodules in control groups was five to nine times more than those in groups containing either zip1246 or zip1290, given similar tumour burden (Fig. 3l). In human lung cancer patient cohorts, gene set enrichment analysis (GSEA) demonstrated a positive correlation between metastasis incidence and miR-1246 or miR-1290 expression in a variety of patient solid tumours that included lung adenocarcinoma (Supplementary Fig. 5). Collectively, our data indicated that miR-1246 and miR-1290 play pivotal roles in mediating the spontaneous metastasis of primary tumour cells. This is consistent with the notion that highly aggressive tumours tend to contain an enriched population of TICs, which can augment tumour growth and metastasis^39^.

### Circulating miRNAs levels correlate with therapy response

**Figure 4:**
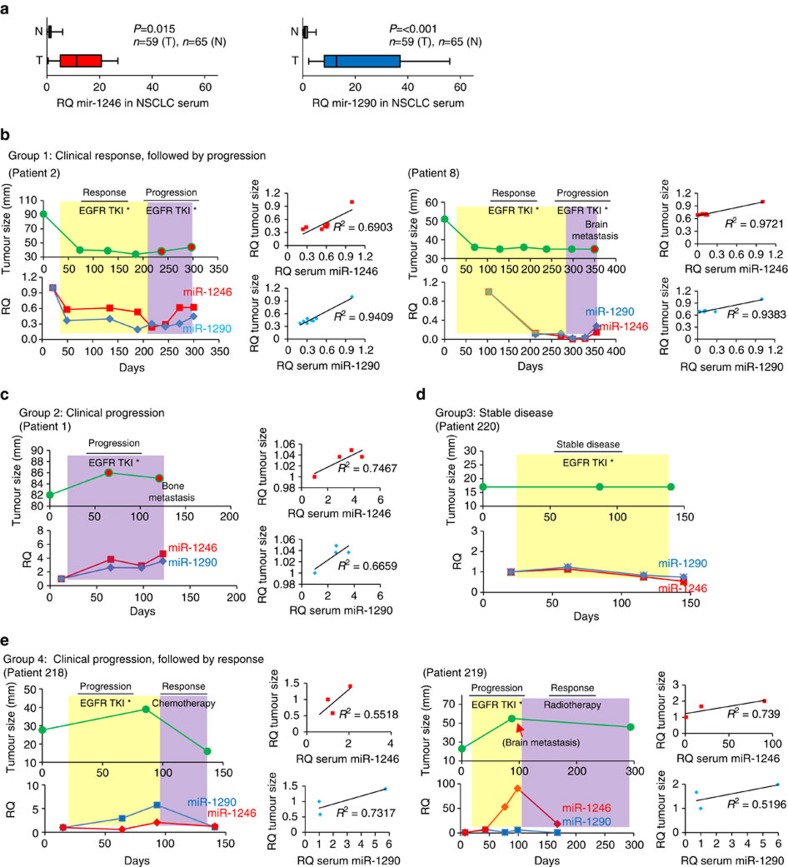
Longitudinal analyses of circulating miRNA levels in response to ongoing therapy in NSCLC patients. (**a**) qRT–PCR analysis of serum miR-1246 and miR-1290 levels in NSCLC patients and healthy individuals. Fold change of miR-1246 and miR-1290 levels is presented as box plot. The median value of serum miR-1246 or miR-1290 levels in healthy individuals was normalized to 1. Data are represented as mean±s.e.m. *P* values were calculated using Student's *t*-tests. T, NSCLC patients (*n*=59); N, healthy individuals (*n*=65). (**b**–**d**) Changes in serum miR-1246 and miR-1290 levels in response to therapy across multiple time points in NSCLC patients. The clinical disease progression status at various times was determined by CT (upper panel). All patients received EGFR TKI *, and in some instances, followed by either chemo or radiotherapy. The circulating miRNA levels are shown in the middle panel. Patients are categorized into four subgroups based on the pattern of clinical response to treatment. Representative response patterns from Group 1 (**b**) (response followed by progression; Patients 2 and 8), Group 2 (**c**) (progression; Patient 1), Group 3 (**d**) (stable disease; Patient 220) and Group 4 (**e**) (progression followed by response; Patients 218 and 219) are shown. The linear association between serum miRNAs levels and tumour size was analysed using Pearson's correlation coefficient (*R*) by SigmaPlot 11. Baseline CT scan was performed at day 0. EGFR TKI*=gefitinib and hydroxychloroquine.

Circulating cell-free miRNAs have been reported in patients harbouring ovarian cancer, melanoma and lymphoma^40^,^41^,^42^. The levels of certain miRNAs appear to be predictive of survival outcomes^26^. In the vast majority of these studies, circulating miRNAs of different cancer patients and normal individuals are compared, typically at a single time point. Furthermore, in instances where circulating miRNA levels were correlated with therapy response, the measurements are obtained from different individuals, thereby confounding analyses. The direct contribution of miRNA levels to disease progression and therapy resistance remains unclear. Here, we performed a longitudinal survey of circulating miR-1246 and miR-1290 in the same individuals to assess variation in their levels in response to ongoing EGFR tyrosine kinase inhibitor (TKI) treatment, which is a standard of care for NSCLC patients with tumours harbouring mutant EGFR. We first examined the serum levels of miR-1246 and miR-1290 from NSCLC patients and healthy individuals (*n*=124) by qRT–PCR. MiR-1246 and miR-1290 levels increased 11.3 times and 12.8 times, respectively, in stage I–III NSCLC patients compared with healthy individuals, as expected (Fig. 4a).

To understand changes in the levels of miRNAs in response to therapy for the same individuals, we recruited a small cohort of late-stage lung cancer patients who were assigned to receive EGFR TKI, and in some cases that progressed on EGFR TKI, with subsequent follow-up radiotherapy or chemotherapy. The baseline levels of circulating cell-free miRNA levels were ascertained before treatment and tracked at several time-points during the course of treatment. On recruitment, tumour sizes on computed tomography (CT) scan were determined using RECIST 1.1. In general, we were able to categorize patients into four subgroups, depending on the pattern of clinical response to treatment, based on either changes in the tumour size or the detection of metastatic disease. Group 1 (*n*=6) comprised of patients who initially responded to therapy, but later progressed as a result of tumour re-growth or occurrence of metastasis. Lung tumours in Patient 2 and 8, for instance, shrank rapidly by as much as 29–56% shortly following therapy, indicating that they were initially responders (Fig. 4b). During this response period, serum levels of miR-1246 and miR-1290 reduced by 69–87% and 63–90%, respectively. For these individuals, however, disease progressed; this is indicated by either tumour re-growth (Patient 2) or brain metastasis (Patient 8) despite continued EGFR TKI treatment. Concomitant increases in the serum miR-1246 and miR-1290 could be detected, thus suggesting their levels to be indicative of the patients' response to therapy. In Group 2 (*n*=3), patients did not respond to therapy from the onset, as determined by continued tumour growth or the detection of metastasis. In these individuals, levels of both miRNAs progressively increased during the course of the disease, resulting in treatment being withdrawn subsequently (Fig. 4c and Supplementary Table 6). Group 3 consisted of a single individual (Patient 220), who had stable disease, and no change in the serum levels of miR-1246 or miR-1290 was detected (Fig. 4d).

In Group 4 (*n*=5), patients initially had disease progression but subsequently responded to therapy. All these patients received EGFR TKI as a first line of treatment, and either chemo- or radiotherapy as the second line of treatment. As an example, Patient 218 progressed on EGFR TKI as tumour grew by 41% on day 86; this was mirrored by increases in miR-1246 and miR-1290 levels. On switching to chemotherapy, the tumour shrank by 59% and the levels of both miRNAs were, similarly, reduced (Fig. 4e). Patient 219, similarly, showed marked tumour growth and occurrence of brain metastasis while on EGFR TKI, but later responded to whole-brain radiotherapy as measured by the reduction in tumour size (Fig. 4e). In this example, the levels of miR-1246 appeared to better mirror the response of patient to therapy than that of miR-1290. Of note, for the vast majority of patients in all four groups, serum levels of miR-1246 and miR-1290 showed a positive correlation with tumour size as well (Fig. 4b,c,e). Thus, our data suggest that serum miRNAs correlate well with the response of patients to ongoing therapy. This strategy of utilizing circulating miRNA levels as a surrogate for assessing disease progression status may, quite possibly, be more sensitive and predictive of metastasis that may not be readily detected in CT scans (Fig. 4b–e and Supplementary Table 6). While CT scans remain diagnostically useful in most instances, they are of limited therapeutic benefit, hence, leading us to explore if targeting miRNAs might be beneficial.

### LNA-targeting miRNAs arrest PDX tumour growth

**Figure 5:**
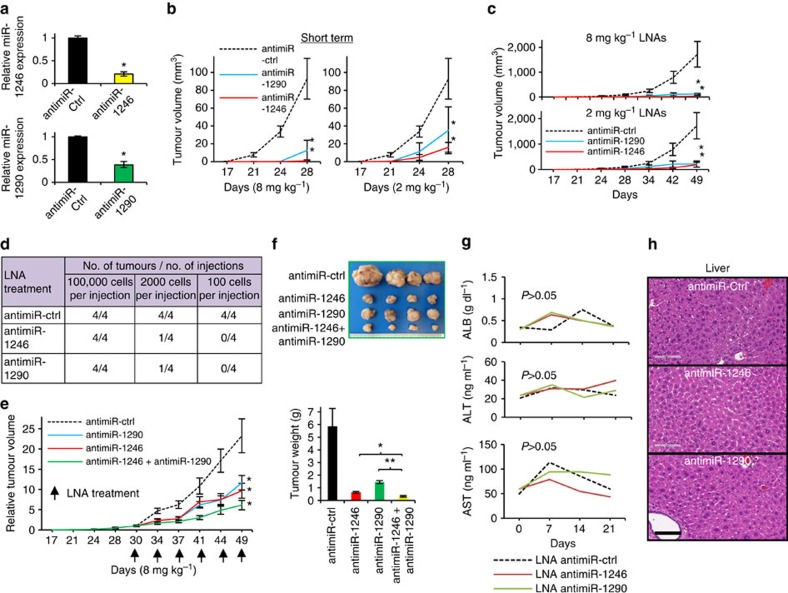
**Administration of anti-miRNA LNA inhibitors** ***in vivo*** **inhibits** tumour **progression.** (**a**) qRT–PCR analysis of miR-1246 and miR-1290 expression in tumoursphere cells on transfection with either LNA-antimiR-1246 or LNA-antimiR-1290. *n*=3. (**b**) Tumour initiation and growth measurements arising from the implantation of tumoursphere cells in NSG mice that were treated with LNA-antimiR-1246 or LNA-antimiR-1290 at either 8 mg kg^−1^ or 2 mg kg^−1^. A total of 100,000 tumoursphere cells were injected subcutaneously on day 0, and LNAs were administrated by intraperitoneal injection twice a week from day 0 to day 28. *n*=4. (**c**) Long-term xenograft tumour initiation and growth arising from the implantation of tumoursphere cells in NSG mice that were treated with LNA-antimiR-1246 or LNA-antimiR-1290. A total of 100,000 tumoursphere cells were injected subcutaneously on day 0, and LNAs were administrated by intraperitoneal injection twice a week from day 0 to day 49 at either 8 mg kg^−1^ or 2 mg kg^−1^. *n*=4. (**d**) Limiting dilution of analysis of tumour initiation from the implantation of tumoursphere cells in NSG mice that were treated with LNA-antimiR-1246 and LNA-antimiR-1290. A total of 100,000, 2,000 and 100 cells were injected subcutaneously on day 0, and LNAs were administrated by intraperitoneal injection twice a week from day 0 to day 90 at 8 mg kg^−1^; *n*=4. (**e**) Effects of LNA treatment on the growth of pre-established tumours in NSG mice. A total of 100,000 tumoursphere cells were injected subcutaneously on day 0, and tumours were allowed to reach 5 mm diameter (∼30 days). Following that, LNAs were administrated by intraperitoneal injection twice a week from day 30 to day 49 at 8 mg kg^−1^. *n*=4. (**f**) Images of xenograft tumours and quantitative analysis of tumour mass formed 19 days after administration of LNAs as shown in (**e**). *n*=4. (**g**) ELISA for serum albumin, ALT and AST levels in mice that were treated with 8 mg kg^−1^ LNA-antimiR-1246 or LNA-antimiR-1290; *n*=4. (**h**) H&E staining of mice liver sections 7 weeks after administration of LNA-antimiR-1246 or LNA-antimiR-1290 at 8 mg kg^−1^. Scale bar, 100 μm. All error bars represent±s.e.m. and statistical significance was calculated using Student's *t*-test; *P<0.05, **P<0.01.

Because tumours appear to depend on miR-1246 and miR-1290 to progress, we reasoned that the inhibition of these miRNAs might impact their growth. We made use of LNA that can be administered into animals and silences specific miRNAs in a highly selective manner. This strategy has been experimentally tested in mice and non-human primates for the treatment of several diseases^43^, and more recently, it has been applied in clinical trials for the treatment of hepatitis C^15^. We first evaluated the utility of LNA against miR-1246 or miR-1290 in cell cultures by transfecting them into lung TICs expressing high levels of both miRNAs. Inhibition of either miR-1246 or miR-1290 significantly reduced their respective expression (Fig. 5a). To assess their therapeutic utility in a physiologically relevant manner, LNAs against either miR-1246 or miR-1290 were introduced intraperitoneally into NSG mice bearing patient-derived lung tumour xenografts. LNA (8 mg kg^−1^) against either miR-1246 or miR-1290 was administered at the same time as lung TIC implantation (1 × 10^5^ cells). This not only delayed the onset of lung TIC-driven tumorigenesis, but also inhibited the long-term growth of xenograft tumours (Fig. 5b,c). Even when a lower dose of 2 mg kg^−1^ was introduced, the delay of tumour initiation and reduction in growth was, similarly, observed (Fig. 5b,c). To directly assess the impact of LNAs on tumour initiation, we treated mice transplanted with limiting dilution number of TS cells. As expected, 100,000 xenografted cells continued to form tumours, albeit smaller and delayed, with LNA administration (8 mg kg^−1^). The LNA, however, completely abrogated tumour-initiation when 100 cells were transplanted, thereby demonstrating the *in vivo* impact of blocking miR-1246 and miR-1290 on tumour initiation (Fig. 5d). To ascertain the impact of anti-miR-1246 and anti-miR-1290 LNA on pre-existing tumours, we first allowed tumours to form (5 mm in length) before treating mice with 8 mg kg^−1^ LNA at 3–4-day intervals. The outgrowth of tumours in the LNA-treated mice was largely inhibited, whereas control-treated mice continued to form large tumours, thereby indicating the therapeutic utility of silencing miR-1246 and miR-1290 in established tumours (Fig. 5e,f). Since the loss of each miRNA, on its own, was capable of reducing the tumorigenic ability of TS cells, we test if the combined inhibition of both miRNAs would demonstrate a more severe impact. Indeed, the concomitant administration of LNAs against miR-1246 and miR-1290 in mice bearing the pre-established tumours could result in a greater inhibition of tumour growth, and suggested the utility of targeting both miRNAs together (Fig. 5f).

A potential confounding challenge with therapeutic agents, including LNA, is animal toxicity, which can undermine their utility^44^. To understand whether LNA targeting miR-1246 or miR-1290 would produce adverse side-effects in mice, we profiled the dynamic changes in albumin levels, as well as alanine aminotransferase (ALT) and aspartate aminotransferase (AST) activities, in mouse serum at different time points following LNA therapy at 8 mg kg^−1^. The levels of albumin and activities of ALT and AST were, in fact, comparable to the sham-treated animals, thus indicating that the LNA, as a therapeutic agent, did not result in overt or measurable toxicity, at least, to the liver (Fig. 5g). This was further confirmed by the histological examination of liver sections obtained from the LNA-treated mice that did not show observable difference compared with control animals (Fig. 5h). Taken altogether, our results demonstrated that miR-1246 and miR-1290 contribute towards lung cancer progression through acting on TIC populations, and more importantly, we provided a proof-of-concept principle that LNA approaches can be used to impact the behaviour of TICs by targeting miRNAs crucial for their function.

### *MT1G* is a target of miR-1246 and miR-1290 that inhibit TICs

**Figure 6:**
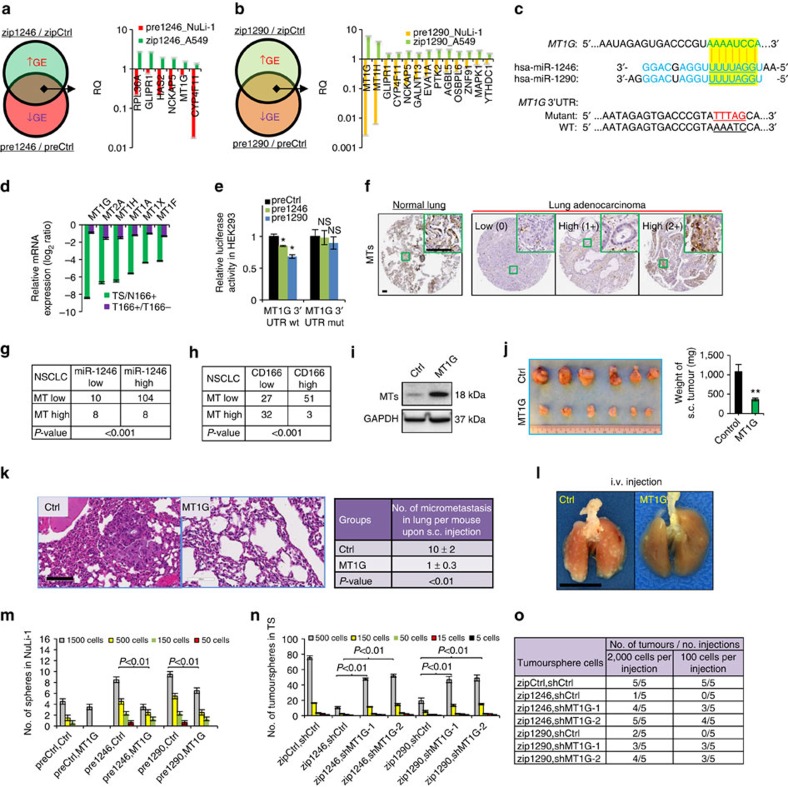
**MT1G, a common target of miR-1246 and miR-1290, inhibits** tumour **growth and metastasis.** (**a**,**b**) Venn diagram showing the identification for putative miR-1246 and miR-1290 target genes and validation by qRT–PCR. Genes (GE) upregulated (↑) on zip1246 or zip1290 in A549 were overlapped with genes downregulated (↓) on pre1246 or pre1290 in NuLi-1; *n*=3. (**c**) Computational prediction of duplex formations between miR-1246 or miR-1290 and the 3′-UTR of *MT1G* mRNA. Mutations generated within the 3′-UTR for the luciferase reported used in **e** are shown in red. (**d**) qRT–PCR analysis of metallothionein in TS and T166^+^ compared with N166^+^ and T166^−^; *n*=3. (**e**) Luciferase activity of wild-type (wt) or mutant (mut) *MT1G* 3′-UTR reporter assay in HEK293 with pre1246 or pre1290; *n*=3. NS, not significant. (**f**) Immunohistochemistry staining of metallothioneins (low: 0; high: 1+–2+) for primary adenocarcinoma and normal lung tissue. Scale bar, 100 μm. (**g**,**h**) The associations between the intensity of metallothioneins expression (immunohistochemistry) and miR-1246 expression (ISH) (**g**) as well as CD166 expression (immunohistochemistry) (**h**) on a NSCLC tissue microarray. *n*=130 and 113 for **g** and **h**, respectively. (**i**) Western blot showing the overexpression of MT1G in TS. GAPDH was loaded as endogenous control. (**j**) Images and quantitative mass of tumours formed 54 days after subcutaneous transplantation of 1 × 10^6^ TS containing MT1G. *n*=6. (**k**) H&E staining and quantification of lung micrometastasis in mice bearing established tumours in **j**; *n*=6. Scale bar, 100 μm. (**l**) Images of lung whole mounts on day 34 following tail-vein injection of 1 × 10^6^ TS bearing MT1G; *n*=5. Scale bar, 1 cm. (**m**,**n**) Limiting dilution analysis of sphere formation in NuLi-1 overexpressing MT1G, and treated with pre1246 or pre1290 (**m**) and in TS bearing shMT1G, and treated with zip1246 or zip1290 (**n**). *n*=3. (**o**) Limiting dilution analysis of tumour formation 60 days after subcutaneous transplantation of 100 and 2000 TS bearing shMT1G, and treated with either zip1246 or zip1290. *n*=5. All error bars represent±s.e.m. For **e**,**j**,**k**,**m**,**n**, statistical significance was calculated using Student's *t*-test; for **g**,**h**, *χ*^2^-test. *P<0.05, **P<0.01.

Because miRNAs are well-known to regulate the activities of downstream targets, which in turn, control the behaviour of a cell, we sought to identify genes that might be directly targeted by miR-1246 or miR-1290. To do so, we performed whole-transcriptome analyses after knocking down miR-1246 or miR-1290 in A549, a metastatic lung cancer cell line, which expressed high levels of the miRNAs, as well as after overexpressing miR-1246 or miR-1290 in NHBE, a normal lung epithelial cell line not expressing the miRNAs. The upregulated genes on knockdown were intersected with genes downregulated on overexpression for each miRNA perturbation to gather a list of candidate mRNAs that could potentially be regulated by either miRNA. We further validated these targets by qRT–PCR (Fig. 6a,b). Genes repressed by miR-1246 include *PRL36A*, *GLIPR1*, *HAS2*, *NCKAP5*, *MT1G* and *CYP4F11*, whereas miR-1290 represses *MT1G*, *MT1H*, *GLIPR1*, *CYP4F11* and *NCKAP5*, among others. A few of the top target genes, such as *MT1G* and *GLIPR1* were, in fact, common targets of both miR-1246 and miR-1290, thus suggesting that they may play an important role in mediating the function of lung cancer cells. Because the regulation of these gene expressions might be attributed to the indirect, secondary effects of miRNA perturbations, a computational approach to validate the interaction between miRNAs and their targets was taken. Using seed sequence base-pairing analyses, we detected the duplex formations between human miR-1246 and miR-1290 with the 3′-untranslated region (UTR) of mRNAs that include *MT1G* (Fig. 6c), thereby highlighting the propensity of both miRNAs to target a common gene.

We chose to focus on understanding the role of MT1G in lung TICs for several reasons. First, MT1G belongs to the metallothionein family of cysteine-rich metalloproteins which bind heavy metals. Metallothionein expression was reduced in several types of cancers, including lung cancer and hepatocellular carcinoma^45^,^46^. MT1/2-knockout mice manifested an increased propensity for carcinogenesis^47^,^48^. Second, in a cohort of NSCLC patients with paired tumour and normal tissues (*n*=9), *MT1G* was strongly expressed in normal tissues but markedly reduced in tumours (Supplementary Fig. 6a). Third, transcriptome analysis comparing tumourspheres and CD166^+^ tumour cells with normal CD166^+^ lung epithelial cells and CD166^−^ tumour cells clearly placed *MT1G* among the top downregulated genes^27^ (Supplementary Fig. 6b,c). Of genes in the metallothionein family, *MT1G* was the most highly suppressed in lung TICs (Fig. 6d).

To confirm *MT1G* as a direct target of miR-1246 and miR-1290, we cloned its wild-type 3′-UTR, as well as made point mutations, and tagged them to a luciferase reporter vector. On co-transfection of either miR-1246 or miR-1290 together with wild-type *MT1G* 3′-UTR reporters into HEK293 cells, the luciferase activities were reduced significantly (Fig. 6e). No change was observed for mutant *MT1G* 3′-UTR reporter. Conversely, knockdown of miR-1246 or miR-1290 in tumourspheres increased luciferase activity of wild-type *MT1G* 3′-UTR but not the mutant (Supplementary Fig. 6d).

Similar to the expression patterns of miR-1246, miR-1290 and CD166, we also observed heterogeneous expression of metallothioneins within human lung tumour sections. Here, we assessed the total levels of all metallothioneins because an antibody specific to MT1G was not available. While metallothioneins were abundant in normal lung tissues, their expression were varied across different adenocarcinomas, ranging from low to high (Fig. 6f and Supplementary Table 7). Interestingly, well-differentiated tumours tended to express higher levels of metallothioneins, whereas poorly differentiated tumours did not. We then sought to correlate the expression of metallothioneins, which was classified as low or high based on immunohistochemistry, with either miR-1246 or CD166 protein level in a cohort of patient tumours. metallothioneins expression was inversely correlated with both miR-1246 (Fig. 6g) and CD166 expression (Fig. 6h), providing an indication that metallothionein expression was repressed in lung TICs which tend to express the miR-1246 and CD166.

The above data indicated MT1G might have a role in the inhibition of tumour-initiation or metastasis. To test this, MT1G was first overexpressed in lung tumourspheres by lentiviral infection (Fig. 6i). Their abilities to form colonies on adherent cultures and on soft agar were inhibited markedly on MT1G overexpression (Supplementary Fig. 7a,b). Their migration (Supplementary Fig. 7c) and invasion capabilities *in vitro* (Supplementary Fig. 7d) were, similarly, impaired. When patient tumourspheres overexpressing MT1G were xenografted subcutaneously into NSG mice, their growth were inhibited by at least threefold relative to control cells (Fig. 6j). Microscopic lung metastases arising from MT1G-expressing tumourspheres were also reduced significantly, as expected (Fig. 6k). To further test whether MT1G controls metastasis independent of tumour size, we introduced MT1G-expressing tumourspheres or control cells directly into the lung through tail-vein injection. The number of metastatic nodules was indeed reduced in the mice injected with MT1G-expressing tumourspheres relative to control cells (Fig. 6l). Interestingly, in mice bearing xenografted tumours that were treated with anti-miR-1246 or anti-miR-1290 LNAs (Fig. 5e), these small residual tumours had increased metallothionein protein level (Supplementary Fig. 7e). It remains, however, unclear whether these contained differentiated non-TICs that are resistant to the TIC-specific LNAs, or the inhibition of miRNAs de-repressed the inhibition of MT1G expression. Clinically, in NSCLC patients, lower metallothionein protein expression was associated with regional lymph node invasion (*P*=0.025 by *χ*^2^-test) and distant metastasis (*P*=0.047 by *χ*^2^-test; Supplementary Fig. 7f). These results are consistent with previous findings on the function of metallothioneins in other cancers^49^,^50^.

If MT1G was a major target of both miRNAs, we tested whether MT1G overexpression could counter the effect of miR-1246 or miR-1290 expression. We first treated NuLi-1 cells with pre1246 or pre1290 and this led to an increase in their sphere-forming ability, as expected (Fig. 6m). However, in a separate experiment, when the cells were simultaneously overexpressing MT1G, their sphere-forming ability was inhibited. Conversely, we also investigated the mechanistic regulation of MT1G and the miRNAs in TS cells. Knocking down either miR-1246 or miR-1290 markedly reduced the tumoursphere-forming ability of TS cells as anticipated (Fig. 6n). The concomitant depletion of MT1G in miR-1246- or miR-1290-depleted cells could, at least in part, rescue the effect of miRNA loss. Finally, the transplantation of these treated cells in limiting cell numbers into NSG mice clearly showed that the TS cells containing MT1G knock down remain tumorigenic even in the presence of miR-1246 or miR-1290 loss, thus confirming MT1G to be their major target (Fig. 6o). Taken altogether, our results indicate that miR-1246 and miR-1290, which are enriched in TICs, have critical roles in regulating tumour growth and metastasis, in part, through the repression of metallothioneins, especially MT1G.

## Discussion

An attractive notion for the more effective treatment of cancer is the use of combination therapies which target specific cell populations within a heterogeneous tumour^51^,^52^,^53^,^54^. Mounting evidence has pointed to TICs as being crucial drivers of tumour initiation, growth, metastasis and resistance^4^,^5^,^55^,^56^,^57^, and one needs to ablate TICs in addition to treating bulk tumour cells^58^,^59^,^60^. Using an unbiased approach to uncover TIC-specific miRNAs from patient-derived tumour cells and tumourspheres, we identified a miRNA signature containing two miRNAs, miR-1246 and miR-1290, both of which contribute towards tumour initiation and metastasis. Although miR-1246 and miR-1290 expression are restricted to TICs, it remains unclear whether they are also associated with tumour grades. In one small cohort of NSCLC patients (*n*=143, National University Hospital), the miRNA expressions appear correlated to tumour stage (Supplementary Tables 3 and 4). However, in another cohort of patients (*n*=397, TCGA data set, Supplementary Fig. 1f), there is no clear correlation (Supplementary Fig. 1g). Thus far, very little information is known regarding the link between TIC frequency with tumour grade, metastasis, therapy resistance or clinical outcome. This is an important question that could shed more light on the biological significance of TIC abundance to disease processes.

While we identified the putative tumour suppressor, *MT1G*, as a common target for miR-1246 and miR-1290, one could not exclude the possibility that other miRNA target genes could also contribute towards the inhibition of tumorigenicity. Since miRNAs are well-known to exert pleiotropic effects on a variety of gene targets, it is arguably more effective to inhibit miRNAs, owing to their potential for silencing other additional endogenous target mRNAs, as in the case of miR-1246 and miR-1290. Previous studies have suggested MT1G to be involved in tumour suppression in colorectal cancer, hepatocellular carcinoma and papillary thyroid carcinoma, where its loss of expression appears to be epigenetically regulated such as through promoter hypermethylation^46^,^61^,^62^,^63^. MT1G also contributes towards chemoresistance. Overexpression of the MT1G isoform sensitizes colorectal cell lines to the chemotherapeutic agents in part through enhancing p53 and repressing NF-κB activity, but the precise connection for the mechanistic role of MT1G leading to the activation of p53, or in regulating other pathways, remains largely elusive^61^.

Because miR-1246 and miR-1290 can confer tumorigenic properties, we sought to determine whether they might also be found enriched in other cancers, compared with their corresponding normal tissue. Interestingly, across the major types of cancers that include breast, colon, and head and neck, both miRNAs are strongly upregulated in tumours relative to their normal counterparts (Supplementary Figs 8 and 9 and Supplementary Tables 2 and 8–10). It is, however, unclear if these miRNAs are restricted to the TIC compartments of these tumours, or could have broader roles in bulk tumour cell populations as well. Nonetheless, our observations indicate miR-1246 and miR-1290 can behave as non-invasive biomarkers that may be exploited for the early detection of a broad spectrum of cancers (Supplementary Fig. 10). We noted from our normal tissue microarray data that the skin, oesophagus, nerve and liver were main tissue compartments that also expressed these miRNAs; this raises the possibility of toxic side-effects on these tissues (Supplementary Fig. 8a–c). However, we did not observe alteration to the skin, changes in appetite or the body weight of mice treated with the LNAs. Notwithstanding, the pharmacodynamic and pharmacokinetic properties of anti-miR LNAs will need to be more rigorously evaluated for assessing their potential as a viable therapeutic strategy.

## Methods

### Patients and samples collection

Eligible patients were pathologically confirmed with the diagnosis of NSCLC. The solid tumours or serum were collected from patients according to protocols approved by the Ethics Committee of the National University of Singapore. Informed consent was obtained from the patients. Serum from stage I–III patients with NSCLC and healthy individuals was collected for serum miRNA profiling. To correlate serum miRNAs levels with therapy response, NSCLC patients at stage IIIB or IV, recruited as part of the NCT00809237 clinical study, took both 250 mg gefitinib, in combination with 600 mg hydroxychloroquine orally once daily, from the start of treatment to documented progression on CT imaging. Blood was drawn at every 4 weeks during treatment. CT was performed and documented in a blinded manner to monitor response to treatment according to the Response Evaluation Criteria in Solid Tumors (RECIST)^64^. This study has been approved by the National Healthcare Group Ethics committee (NHG IRB).

### Cell lines

The A549 and HEK293 cell lines were obtained from ATCC and cultured in DMEM (high glucose; GIBCO) with 10% FBS, 2 mM L-glutamine and 1% penicillin–streptomycin. Primary NHBE and SAEC were obtained from Lonza and maintained in BEGM or SAGM complete-growth medium (Lonza). Immortalized NuLi-1 cell line was obtained from ATCC and cultured in BEGM serum-free complete-growth medium (Lonza) supplemented with 50 μg ml^−1^ G418 (Sigma).

### Isolation of single tumour cells

All patients were first diagnosed with primary NSCLC without other tumour occurrences. They did not receive any therapy before surgery. Samples were chopped into small pieces, and incubated in 1 mg ml^−1^ collagenase/dispase solution (Roche, Indianapolis, IN) with 0.001% DNAse (Sigma-Aldrich, St Louis, MO) and 2% antibiotics (Sigma) in a water bath at 37 °C for 3 h. After incubation, the suspensions were passed through 70- and 40-μm cell-strainers (BD Falcon, San Jose, CA) and centrifuged at 122*g* for 5 min at 4 °C. Cells were then resuspended in red blood cell lysis buffer (eBioscience, San Diego, CA) for 4 min at room temperature with intermittent shaking. The cell viability was evaluated by trypan blue dye exclusion. Live single cells accounted for 90% of the whole population.

### Antibodies

For fluorescence-activated cell sorting by flow cytometry, primary mouse anti-human CD166^−^ phycoerythrin (FAB6561P) was derived from R&D and anti-human EPCAM-FITC was derived from Miltenyi Biotech. For immunohistochemistry staining, primary rabbit anti-human E-Cadherin (1:500, cat #1702-1) was from Epitomics. Primary mouse anti-CD166 (1:50, clone MOG/07, NCL-CD166) was purchased from Novocastra (Leica Biosystem). Primary anti-human metallothionein (1:50, clone E9, M0639) was from Dako. Secondary goat anti-mouse antibody conjugated with horseradish peroxidase (HRP; 1:100, cat #P0447) and goat anti-rabbit antibody conjuagated with HRP (1:100, cat # K4003) were from Dako. For western blot, primary rabbit anti-metallothionein antibody (1:300, clone FL-61, sc-11377) was from Santa Cruz. Mouse anti-GAPDH (1:5,000, Santa Cruz, sc-47724) and rabbit anti-α tubulin (1:2,000, Abcam, ab4074) were used as loading control. Secondary goat anti-mouse IgG H&L (HRP; 1:10,000, ab6789) and goat anti-rabbit IgG H&L (HRP; 1:10,000, ab6721) were purchased from Abcam. For ISH, sheep anti-digoxigenin (DIG)—alkaline phosphatase (AP; cat #11093274910) came from Roche.

### Fluorescence-activated cell sorting

For sorting, single-cell suspension was incubated with FcR blocking reagent (Miltenyi Biotech) in ice for 20 min. Then the cells were incubated with antibody against CD166 conjugated with phycoerythrin (R&D), and antibodies against lineage markers (human CD45 and CD31). To exclude dead cells, 7-amino-actinomycin D (BD PharminGen) was added before sorting. Appropriate isotype antibodies were used as controls.

### Plasmids

The *MT1G* 3′-UTR sequence was cloned into the pEZX-MT01 firefly/Renilla Duo-Luciferase reporter vector (GeneCopoeia). The pmiRZip-1246 and pmiRZip-1290 in miRZip-copGFP lentiviral vector (System Biosciences) to stably knockdown miR-1246 or miR-1290 expression was used following the manufacturer's instructions and contained the following shRNA sequence: 5′- AAUGGAUUUUUGGAGCAGG -3′ or 5′- UGGAUUUUUGGAUCAGGGA -3′, respectively. The pmiR-1246 and pmiR-1290 in pCDH-CMV-MCS-EF1-copGFP (CD511B-1) lentiviral vector (System Biosciences) to stably overexpress miR-1246 or miR-1290 was used following the manufacturer's instructions and contained the following sequence: 5′- AAUGGAUUUUUGGAGCAGG -3′ or 5′- UGGAUUUUUGGAUCAGGGA -3′, respectively. Precision pLOC-LentiORF-MT1G (OHS5899, Open Biosystems) and empty vector control Precision pLOC-LentiORF RFP (OHS5833) were used to overexpress *MT1G*. pTRIPZ inducible lentiviral shRNA against *MT1G* and pTRIPZ empty vector were used to knockdown *MT1G*. The sequences for sh*MT1G*-1 and sh*MT1G*-2 are 5′- TATTATTCACATATTTCAC -3′ and 5′- TTTTGCACTTGCAGGAGCC -3′, respectively. LNA inhibitors against miR-1246 or miR-1290 (Exiqon) contained the following sequence: 5′- TGCTCCAAAAATCCAT -3′ or 5′- CCTGATCCAAAAATCC -3′, respectively, and the scramble LNA inhibitor control's sequence was: 5′- ACGTCTATACGCCCA -3′.

### Transient transfection and luciferase assay

PureFection (System Biosciences) was used for transient transfection. In all, 100 ng of wild-type or mutant 3′-UTR reporter constructs of *MT1G* constructs (GeneCopoeia) were cotransfected with 100 ng of pCDH-miR-1246, pCDH-miR-1290, pmiRZip-1246, pmiRZip-1290 or scrambled control vectors into HEK293 or tumoursphere cells. Firefly and Renilla luciferase activities were measured 48 h post-transfection using dual-luciferase reporter system (Promega). The firefly luminescence was normalized to Renilla luminescence as an internal control for transfection efficiency. MiR-1246- or miR-1290-binding site 5′- AAATC -3′ was substituted with 5′- TTTAG -3′ in mutated *MT1G*.

### Microarray assay

Agilent Human miRNA Microarray Release 16.0, 8 × 60 K (G4872A-031181, Agilent Technologies) was used to identify miRNAs expressed in lung TICs. Total RNA (100 ng per sample) was hybridized to the microarrays. We compared the miRNA expression profiles of tumour spheres, NSCLC patient-derived CD166^+^ and CD166^−^ xenograft tumour cells from three NSCLC patients, as well as two normal lung epithelial cells, including NHBE and SAEC. MiRNA labelling, hybridization washing, scanning, feature extraction and application were carried out according to the manufacturer's instructions. All miRNA raw data were normalized based on the cross-correlation method^65^. Significantly changed miRNAs were identified based on average fold change cutoff of 1.5 and the cutoff of the *P* value cross all replicates at 0.05.

HumanHT-12 v4 Expression BeadChip (Illumina) was used to identify the genes upregulated in A549 knocking down miR-1246 or miR-1290, and genes downregulated in NuLi-1 overexpressing miR-1246 or miR-1290. Total RNA (200 ng per sample) was hybridized to the microarrays. Total RNA was converted to double-stranded cDNA, followed by an amplification step to generate labelled cRNA. The follwoing hybridization, image processing and raw data extraction were performed according to the manufacturer's instructions. All mRNA raw data were normalized based on the cross-correlation method^65^. Significantly changed mRNAs were identified based on average fold change cutoff of 1.5 and the cutoff of the *P* value cross all replicates at 0.05.

### MiR-1246 and miR-1290 targets prediction

Potential miR-1246 and miR-1290 targets were identified by using two sets of wide-transcriptome microarray profiles: NuLi-1 relative to NuLi-1 cells overexpressing miR-1246 or miR-1290 (Illumina humanHT12_V4), and A549 relative to A549 cells knocking down miR-1246 or miR-1290 (Illumina humanHT12_V4). The following criteria were used to identify the possible miR-1246 or miR-1290 target genes: (1) genes downregulated >1.5-fold on miR-1246 or miR-1290 overexpression in NuLi-1 cells, and (2) genes upregulated >1.5-fold on miR-1246 or miR-1290 downregulation in A549 cells. All potential targets were subsequently verified by qRT–PCR.

### Isolation and quantification of circulating tumour miRNAs

Whole blood was collected in red-topped tubes (BD). Blood was clotted by leaving it undisturbed at room temperature for 30 min. The clot was removed by centrifugation at 1,000*g* for 10 min in a refrigerated centrifuge. Then the serum in the upper supernatant was transferred immediately into a clean tube for circulating miRNA assays.

### Real-time PCR for miRNAs and genes

Total RNA was extracted from solid tissues and cultured cells using mirVana miRNA Isolation Kit (Ambion) as well as from serum using mirVana PARIS kit (Ambion) according to the manufacturer's instructions. MiRNA expression was assessed by Taqman MicroRNA assay, and the gene expression of mRNAs was evaluated by Taqman Probes (Applied Biosystems). Taqman miRNA probes were as follow: hsa-miR-1246 (462575_mat), hsa-miR-1290 (002863), hsa-miR-130a (000454), hsa-miR-130b (000456), hsa-miR-196a (241070_mat), hsa-miR-196b (002215), hsa-miR-630 (001563), hsa-let-7b-5p (002619), hsa-let-7c (000379), hsa-let-7d-5p (002283), hsa-let-7i (002221), hsa-miR-106b (000442), hsa-miR-125b (000449), hsa-miR-23a (000399), hsa-miR-25 (000403), hsa-miR-320c (241053_mat), hsa-miR-3667-5p (462350_mat), hsa-513-5p (002090), hsa-miR-9* (002231). Taqman gene-expression probes were as follow: *MT1G* (Hs02578922_gH), *MT1H* (Hs00823168_g1), *GLIPR1* (Hs01564143_m1), *HAS2* (Hs00193435_m1), *EVA1A* (Hs00259924_m1), *CYP4F11* (Hs01680107_m1), *PRL36A* (Hs01586542_g1), *OSBPL6* (Hs00992951_m1), *MAPK1* (Hs01046830_m1), *YTHDC1* (Hs00180158_m1), *AGBL5* (Hs00222447_m1), *ZNF91* (Hs00602754_mH), *PTK2* (Hs01056457_m1), *NCKAP5* (Hs00418350_m1), *GALNT13* (Hs00287613_m1). MiRNA expression was normalized to that of hsa-RNU48 (1006), miR-16 (000391), miR-26b (000407) and miR-92 (000430) (solid tissues), or hsa-miR-16 and hsa-miR-374 (000563) (cultured cells), or hsa-miR-425-5p (001516), hsa-RNU-48 and hsa-miR-16 (serum samples). Gene expression was normalized to *GAPDH*. Each sample was run in triplicate for real-time PCR.

### Sphere-formation assay

Single cells were resuspended in complete serum-free media. It contains DMEM/F12 with 50 ng ml^−1^ epidermal growth factor (Invitrogen), 20 ng ml^−1^ basic fibroblast growth factor (Invitrogen), 0.4% bovine serum albumin (Sigma), 0.05 mg ml^−1^ insulin–transferring–selenium and 1% MEM non-essential amino acid (Gibco). Then cells were plated at 10,000 cells per well in six-well non-treated cell culture plates (Nunc). Fresh medium was replenished every 3 days. Tumourspheres were cultured for 10–14 days and then quantified. For passaging, tumourspheres were digested into single cells using accutase (Chemicon) and re-plated. For limiting dilution assay, 50, 150 and 500 of single cells were plated to assess sphere formation.

### Colony-formation assay in plate and soft agar

Single cells were plated in 10-cm dishes in triplicates with 1,000 cells per dish. Fresh medium was replenished every 3 days. The cells were incubated for 10 days followed by Giemsa (Sigma) staining. The plates were air-dried and photographed, and the total number of colonies was analysed by openCFU ( http://opencfu.sourceforge.net)^66^.

For soft-agar colony formation, 500 live cells were mixed with 0.35% top-agar and were plated onto 0.6% base-agar in six-well plates with triplicates. The cells were incubated for 14–21 days followed by INT staining overnight. The plates were photographed and the colony numbers were counted by Gelcount (Oxford optonix).

### Cell proliferation assay

Cultured cells were plated into 96-well plates with 400 HEK293 cells per well in four replicates on day 0. The cell viability was measured every day using CellTiter-Glo luminescent cell viability assay kit (Promega) according to the manufacturer's instructions. Luminescent signal was recorded as relative light units. The relative proliferation index on day 0 was normalized as 1.

### Cell migration and invasion assays

*In vitro* cell migration and invasion assay were performed using Boyden chambers (BD bioscience) that use 8 μm micropore membranes without Matrigel (for migration assay) or with Matrigel (for invasion assay). Both assays were carried out according to the manufacturer's instructions. The cells were resuspended in 0.1% bovine serum albumin in DMEM/F12 medium and seeded in the upper chamber at a concentration of 1 × 10^5^/0.5 ml. The chambers were incubated in the wells containing DMEM/F12 medium with 10% FBS for 6 or 24 h. Filters were fixed with 3.7% formaldehyde and stained with Giemsa. The cells on the upper surface of the filters were removed by swabbing with a cotton swab, and the cells that had migrated to the reverse side were counted in 10 random fields under a microscope (Zeiss) at 400 × magnification.

### Lentiviral-mediated miRNA and MT1G overexpression or knockdown

Lentivirus was produced in 293FT packaging cells and collected 48–72 h post-infection. For lentiviral overexpression or knockdown of miR-1246 or miR-1290, cells (tumoursphere, A549, NuLi-1 and HEK293) were infected with the lentiviral supernatant for 48 h in the presence of 8 μg ml^−1^ polybrene (Sigma). Two days after infection, puromycin was added to the media at 1 μg ml^−1^, and cell populations were selected for 1–2 weeks. For lentiviral overexpression of *MT1G*, cells (tumoursphere and HEK293) at 70% confluence were transduced with *MT1G* lentiviral particles (1.64 × 10^9^ TU ml^−1^, Open Biosystems) or control lentiviral particles (2 × 10^8^ TU ml^−1^, Open Biosystems) together with polybrene. Then the infected cells were passaged and selected by blasticidin S (Invitrogen) at 12 μg ml^−1^ for 1–2 weeks. For inducible lentiviral knockdown of *MT1G*, tumoursphere cells at 70% confluence were transduced with two shRNAs against *MT1G* lentiviral particles (Open Biosystems) or control lentiviral particles together with polybrene. Then the infected cells were passaged, induced by 0.5 ug ml^−1^ doxycycline and then selected by puromycin at 1 μg ml^−1^ for 1–2 weeks.

### Western blot

Cells were collected and lysed with Nonidet-P40 supplemented with protease inhibitor cocktail (Roche). Protein concentrations of the extracts were measured using BCA assay (Pierce) and equalized with the extraction reagent. Equal amount of the extracts was loaded and subjected to SDS-PAGE, transferred onto nitrocellulose membranes. Peroxidase-conjugated anti-mouse (1:10,000, ab6789) or rabbit IgG (1:10,000, ab6721) was used as secondary antibody and the antigen-antibody reaction was visualized by Amersham ECL Prime detection reagent (GE Healthcare). Uncropped Western Blot images are included in Supplementary Fig. 11.

### H&E and immunohistochemistry

Samples were formalin-fixed, paraffin-embedded (FFPE), sectioned and stained with haematoxylin-eosin (H&E) according to standard histopathological techniques. For immunohistochemistry, sections were incubated with anti-human CD166 (Novaocastra), E-cadherin (Epitomics) and metallothioneins (Dako), and visualized using the Envision HRP Polymer System (Dako). All images were captured on a high-throughput Leica SCN400 scanner.

### MiRNA ISH

ISH on tissue microarray sections from FFPE tissue sample with human lung and other types of cancers was applied by using the miRCURY LNA microRNA ISH Optimization kit 5. Five micrometer-thick tissue sections were incubated with 15 μg ml^−1^ proteinase K for 45 min at 37 °C. After washing, the sections were incubated with 20 nM LNA probes (5′-double-digoxigenin-labelled LNA probes specific for human miR-1246 (5′- cctgctccaaaaatccatt -3′), miR-1290 (5′- tccctgatccaaaaatcca -3′) or scrambled probe (5′- gtgtaacacgtctatacgccca -3′) (Exiqon)) in hybridization buffer (Roche) overnight at 55 °C. After stringent washes, sections were blocked with 2% sheep serum and further incubated with sheep anti-digoxigenin AP (Roche; 1:500) at room temperature for 2.5 h. Sections were washed in PBS-T (0.1%) and miRNA-bound LNA probes were detected by AP substrate (Roche) at room temperature for 1.5 h. After counterstaining with Nuclear Fast Red (Vector laboratories), slides were mounted using mounting medium (Eukitt). Image acquisition was performed with high-throughput Leica SCN400 scanner and/or Olympus FluoView FV1000. LNA 5′-digoxigenin-labelled (5′- cacgaatttgcgtgtcatcctt -3′) U6 snRNA probe at 0.1 nM was used as positive control.

For co-localization of miRNA-1246/miR-1290 and CD166 protein in FFPE tissues, the sections were stained with LNA probe by ISH followed by immunohistochemistry with anti-CD166 (Novaocastra).

### Tissue microarray

A tissue microarray with regional lymph nodes, malignant and cancer-adjacent normal lung specimens from NSCLC patients was constructed. Tumour specimens were transferred to the Department of Pathology, National University Hospital of Singapore within 1 h after surgical removal. Suitable areas for tissue retrieval were marked on standard H&E sections, punched out of the paraffin block and inserted into a recipient block. The punch diameter was 0.6 mm. The tissue array was cut in 4-μm thick sections. Tissue microarrays including multiple organs (FDA808b-1, 2 and BC00112) were purchased from Biomax. MiR-1246, miR-1290 or metallothionein staining was independently scored by two anatomical pathologists (M.E.N and Y.H.P). Staining intensity was scored semi-quantitatively (score 0: undetectable; 1+: weak; 2+: moderate; 3+: strong) and grouped as low (score 0) or high (scores 1+∼3+).

### Transfection by LNAs *in vitro*

tumoursphere cells were plated at 3,000 cells in serum-free medium in a 96-well non-treated plates to reach 50–60% confluence. In all, 50 nM of LNA anti-miR-1246, anti-miR-1290 or negative control (Exiqon) with fluorescine and PureFection (System Biosciences) were applied for transfection. The transfected cells were collected after culturing for 40 h.

### Animal studies

All research involving animals complied with protocols approved by the A*STAR Biological Resource Centre Institutional Animal Care and Use Committee. Four to 6-week-old female NSG immunodeficient mice (Jackson Laboratory) were used for subcutaneous injections, and 6–8-week-old female NSG mice were used for tail-vein injections. For subcutaneous xenograft tumour assay and/or spontaneous metastasis assay, 100 to 1 × 10^6^ cells (CD166^+^ and CD166^−^ tumour cells, tumoursphere, HEK293 and NuLi-1) in serum-free medium and Matrigel (BD; 1:1) were inoculated into the flank of NSG mice. The xenograft tumour formation was monitored by calipers twice a week. The recipient mice were monitored and killed when the tumours reached 2 cm in diameter, and thus the metastases by tumoursphere cells were evaluated 60–90 days post transplantation. The subcutaneous xengoraft tumours and the spontaneous metastasis into lung were analysed under a dissecting microscope equipped with GFP fluorescence imaging.

For the tail-vein assay of cancer metastasis, cells were inoculated intravenously into 6–8-week-old female NSG mice, and both the lungs and the liver were removed on the 13th, 27th and 33rd day post transplantation, and fixed with 10% neutral-buffered formalin. Tumour metastasis was recorded by fluorescence imaging. For quantitative analysis of metastasis, the metastatic lung nodules >0.4 mm were counted. Metastatic index was calculated as number of lung nodules per mouse/volume of subcutaneous tumour.

### LNA Synthesis and administration

Custom-made miRCURY LNAs for *in vivo* application were designed and synthesized as unconjugated and fully phosphorothiolated oligonucleotides by Exiqon. The sequences of the LNA targeting miR-1246 or miR-1290 were fully complementary to the mature miRNA sequence: 5′- TGCTCCAAAAATCCAT -3′ (LNA antimiR-1246) and 5′- CCTGATCCAAAAATCC -3′ (LNA antimiR-1290); the scrambled LNA control was 5′- ACGTCTATACGCCCA -3′ (LNA antimiR-ctrl). LNA was intraperitoneally delivered to mouse at a dose of 2 or 8 mg kg^−1^ body weight in 1 × PBS. At the beginning of tumour-formation assay, mice were injected twice a week from day 0 on implantation of 1 × 10^5^, 2,000 and 100 tumoursphere cells via subcutaneous injection and killed 49–90 days after LNA administration. In mice with established subcutaneous xenograft tumours (5 mm in length), LNAs were administrated twice a week at 8 mg kg^−1^ body weight for 19 days. Four mice were used in each group.

### Enzyme-linked immunosorbent assay

Mouse serum albumin, ALT and AST levels were measured at different time points after LNA treatment by enzyme-linked immunosorbent assay according to the manufacturer's instructions (USCN).

### Data analysis

For survival analysis and GSEA, two lung tumour (LUAD and LUSC) RNA-seq data sets were utilized from the TCGA data portal. The survival analysis was based on the Kaplan–Meier method for two sample groups of low and high miRNA expression. In segragation of patient samples into high and low groups, normalization using the total numbers of mappable reads across all samples was first performed. Then the miRNA mean expression as cutoff was applied to segregate high- and low-expression samples. In addition, those middle samples with expression close to the mean expression value were removed, since they might be equally classified into either group. For identification of enriched gene sets, GSEA was performed based on the normalized data and using GSEA v2.07 tool ( http://www.broad.mit.edu/gsea/) with msigdb.v4.0.

### Statistical analysis

Data are presented as the mean±s.e.m. Unless otherwise stated, statistical significance was determined by a Student's two-tailed *t*-test. *P*<0.05 was considered statistically significant. The associations between expressions of miRNAs or CD166 and metallothioneins were evaluated using *χ*^2^-test. The linear association between serum miRNAs levels and tumour size was analysed using Pearson's correlation coefficient (*R*) by SigmaPlot 11.

## Additional information

**Accession codes**: The microarray data can be found in the Gene Expression Omnibus (GEO) with accession number GSE69631.

**How to cite this article:** Zhang, W. C. *et al*. Tumour-initiating cell-specific miR-1246 and miR-1290 expression converge to promote non-small cell lung cancer progression. *Nat. Commun.* 7:11702 doi: 10.1038/ncomms11702 (2016).

## Supplementary Material

Supplementary Information Supplementary Figures 1-11 and Supplementary Tables 1-10
