# Supplementary material for: Tumour-initiating cell-specific miR-1246 and miR-1290 expression converge to promote non-small cell lung cancer progression
Source: Nat Commun. 2016 Jun 21;7:11702. doi: 10.1038/ncomms11702 (PMC4919505; doi:10.1038/ncomms11702)
Supplement: Supplementary Information — Supplementary Figures 1-11 and Supplementary Tables 1-10 [file ncomms11702-s1.pdf]

Supplementary Figures

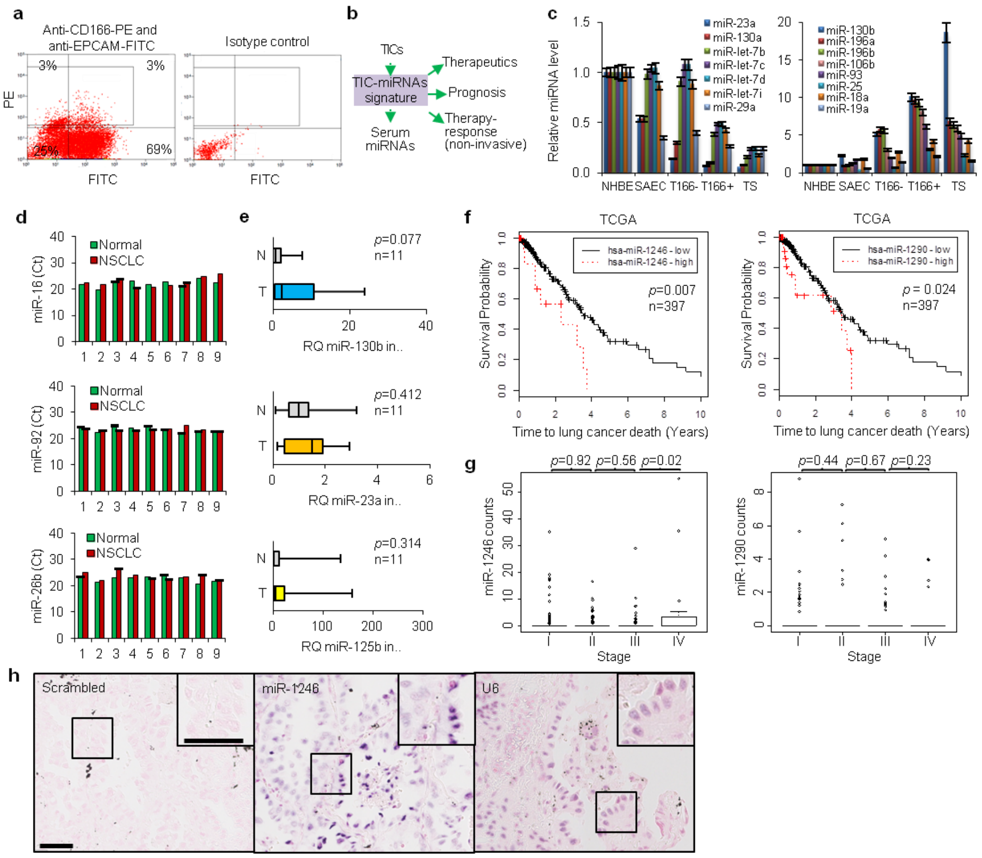

Supplementary Fig. 1. A miRNA signature enriched in TICs in NSCLC. (a) Flow cytometry analysis of CD166 and EPCAM in primary lung tumor (left). Isotype antibodies were applied as control (right). **(b)** Strategy for the identification and characterization of TIC-enriched miRNAs in NSCLC. **(c)** Quantitative RT-PCR analysis of candidate miRNAs listed in Fig. S1b in non-tumorigenic cells (NHBE, SAEC and T166-) and TICs (T166+ and TS) in NSCLC. Both downregulated miRNAs (left panel) and upregulated ones (right panel) are shown. The miRNAs level in NHBE was normalized as 1; n=3 replicates. **(d)** Quantitative RT-PCR analysis of miR-16, miR-92 and miR-26b, which are employed as endogenous controls, across primary NSCLC tumors and normal tissues. Ct, cycle threshold; n=9. **(e)** Quantitative RT-PCR analysis of miR-130b, miR-23a and miR-125b levels, presented as box plot, in paired tumor and normal tissues in NSCLC. The vertical line within the box indicates the median, boundaries of the box indicate the 25th- and 75th -percentile, and the whiskers indicate the highest and lowest values of the results. The median values in normal tissues were normalized as 1. N, normal; T, tumor; RQ, relative quantification; n=11. **(f)** Kaplan Meier curves showing the survival analyses of lung adenocarcinoma patients whose tumors express either high or low levels of miR-1246 and miR-1290 based on TCGA miRNA-Seq data. High or low expression was defined after performing normalization using the total numbers of mappable reads across all samples. TCGA, The Cancer Genome Atlas; n=397. (**g**) Correlation between expression of miR-1246 or miR-1290 and NSCLC stages based on TCGA miRNA-seq data. n=397. **(h)** ISH for miR-1246 in primary lung adenocarcinoma. Both scrambled miRNAs (left) and U6 (right) are applied as negative and positive controls, respectively. Scale bar, 50 µm. All error bars represent ±SEM and statistical significance was calculated using Student's *t*-test (e,g) or log-rank test (f).

a

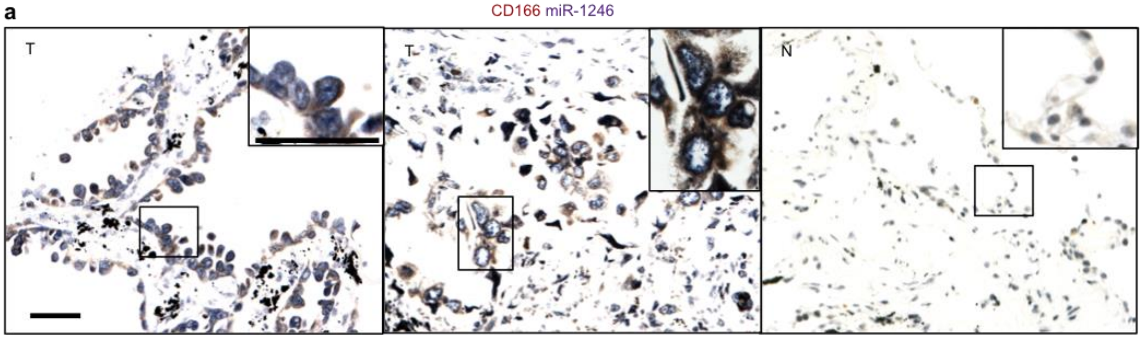

b

NSCLC	miR-1246 low	miR-1246 high
miR-1290 low	21	19
miR-1290 high	14	115
p-value	<0.001	

Supplementary Fig. 2. Expressions of CD166 and miR-1246 in lung tumors and normal tissue. (a) Co-staining CD166 (IHC) and miR-1246 (ISH) in lung tumors and normal tissues. Co-expression of CD166 (in brown) and miR-1246 (in purple) are shown in adenocarcinoma (T) and normal lung (N). Scale bar, 50 µm. **(b)** The association between the intensity of miR-1246 expression and miR-1290 expression by ISH on a NSCLC tissue microarray. *p*-value was calculated using Chi-squared test; n=169.

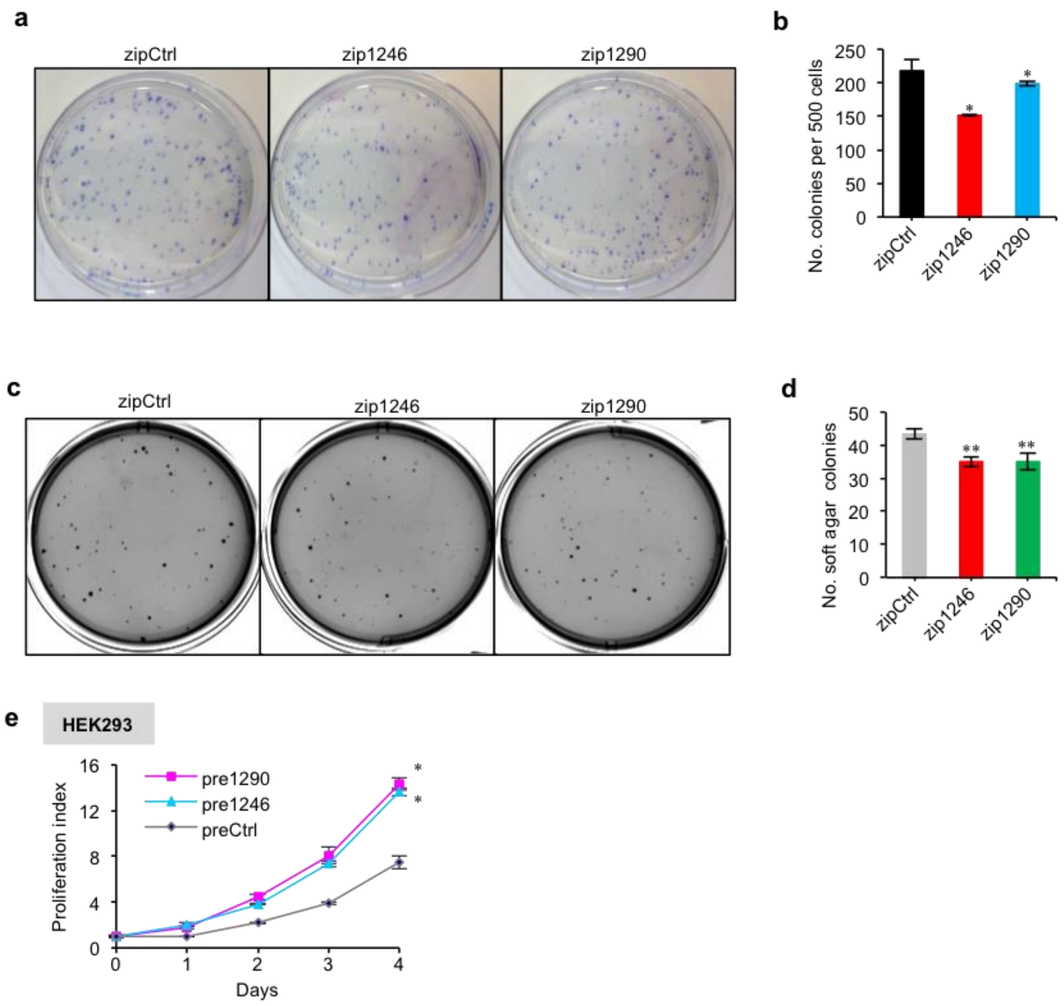

Supplementary Fig. 3 The impact of mir-1246 or mir-1290 perturbations on *in vitro* growth. (a) Colony formation assay in adherent conditions of tumorsphere cells treated with either zip1246 or zip1290. 500 cells were plated. **(b)** Quantitative analysis of the number of colonies formed under adherent conditions in Fig. S3a. n=3 replicates. **(c)** Soft agar colony formation assay of tumorsphere cells that treated with either zip1246 or zip1290. 500 cells were plated. Colonies were stained with INT and counted on day 28. **(d)** Quantitative analysis of soft agar colony formation in Fig. S3c. n=3 replicates. (**e)** Proliferation assay of HEK293 cells treated with either pre1246 or pre1290. 100 cells were plated and the numbers of cells were evaluated on day 1, 2, 3 and 4 by CellTiter-Glo luminescent assay. n=3 replicates. All error bars represent ±SEM and statistical significance was calculated using Student's *t*-test; *p<0.05, **p<0.01.

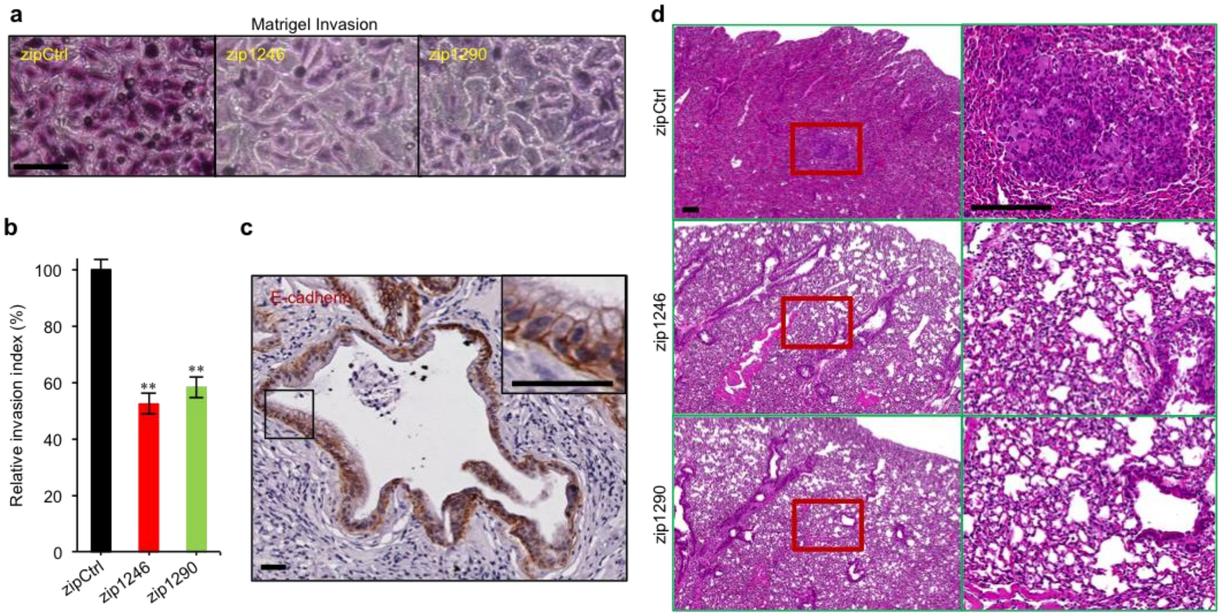

Supplementary Fig. 4. miR-1246 or miR-1290 contribute towards lung cancer invasion and metastasis. (a) Transwell matrigel invasion assay of tumorsphere cells treated with either zip1246 or zip1290. 40,000 cells were seeded. The invaded cells on the lower membrane of transwell inserts were stained with Giemsa and counted 30 hours after plating. Scale bar, 50 µm. **(b)** Quantification of invasion assay as shown in Fig. S4a. n=3 replicates. All error bars represent +SEM and statistical significance was calculated using Student's *t*-test; $^{**}p<0.01$. **(c)** H&E staining for mouse lung sections as shown in Fig. 3k on day 60, following the subcutaneous injection of 1×10^6 tumorsphere cells that were treated with either zip1246 or zip1290. Sections of lung lobes in low (left) and high magnifications (right) are shown. Scale bar, 200 µm. **(d)** IHC staining for E-cadherin in primary lung adenocarcinoma. Staining pattern of E-cadherin (see inset) is shown in higher magnification. Scale bar, 50 µm.

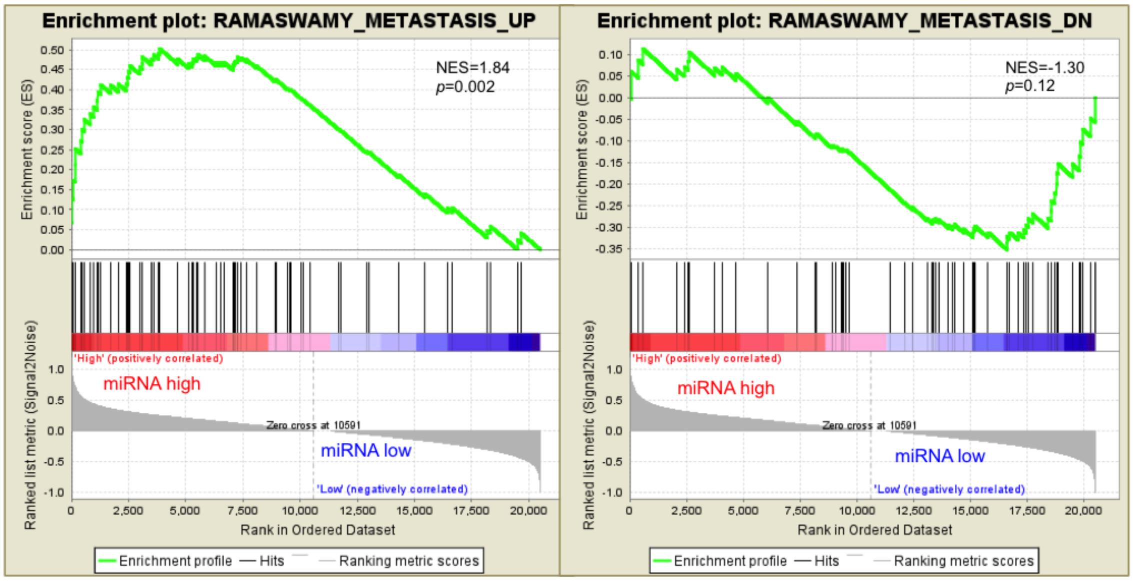

Supplementary Fig. 5. Analysis for metastasis in patients with solid tumors including of lung adenocarcinoma based on the intensity of miRNAs expression in tumors. Heat maps showing miR-1246 and miR-1290 of the Gene Set Enrichment Analysis (GSEA) plots. The *p*-values were calculated with GSEA analysis. NES, normalized enrichment score; DN, down.

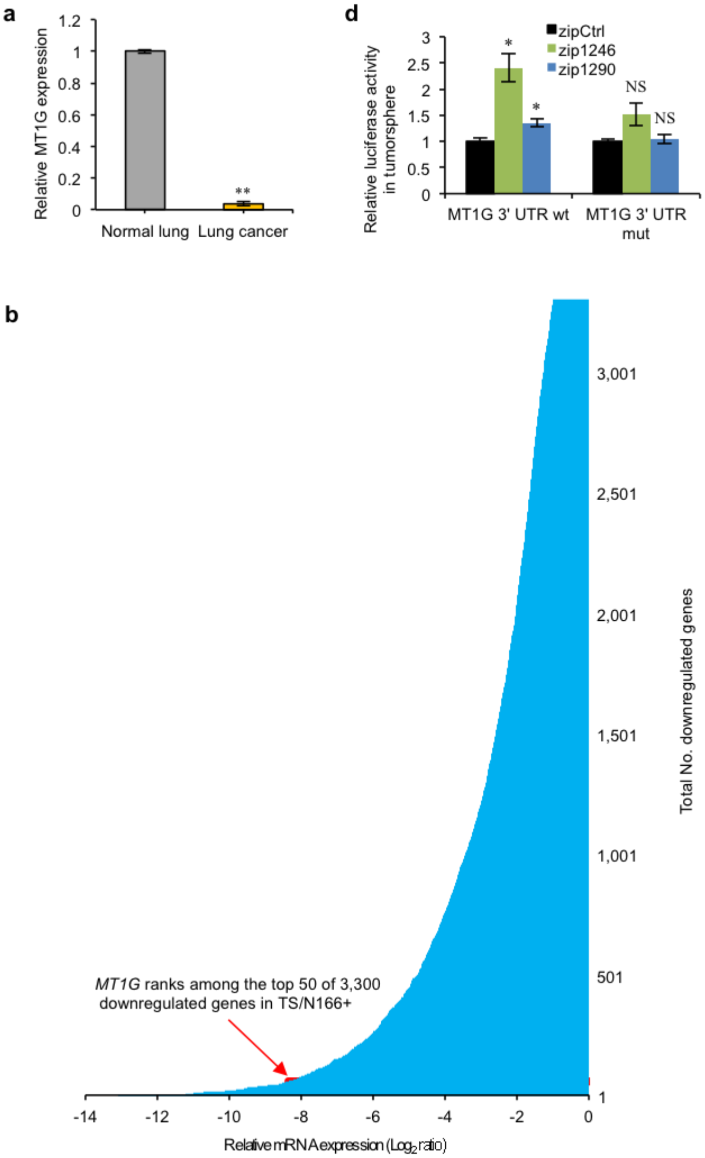

c

SYMBOL	TS/N166+ (log$_2$ ratio)
SFTPC	-13.09
HLA-DRA	-12.47
CCL3L3	-11.99
IGLL1	-11.71
IL1B	-11.21
CD74	-11.20
SCGB1A1	-11.12
SCGB3A1	-11.02
TYROBP	-11.01
C1QB	-11.00
OLR1	-10.91
MGP	-10.90
CCL3	-10.75
HLA-DQA1	-10.72
FCER1G	-10.57
RGS1	-10.53
ALOX5AP	-10.37
MARCO	-10.20
CCL3L1	-10.13
CCL4L1	-10.02
IL6	-9.93
AQP9	-9.85
SFTPD	-9.80
C1QC	-9.76
NAPSA	-9.73
FBP1	-9.71
LAPTM5	-9.62
CECR1	-9.58
CCL2	-9.58
TNF	-9.57
CD52	-9.54
NLRP3	-9.52
CCR7	-9.49
SPARCL1	-9.41
CXCL2	-9.37
C19orf59	-9.31
IGJ	-9.21
HSPA1A	-9.08
SCGB3A2	-9.04
VWF	-8.99
CD163	-8.98
CES1	-8.94
HAVCR2	-8.86
CCL20	-8.85
PTGDS	-8.85
PLEK	-8.72
TACSTD2	-8.64
EBI2	-8.60
**MT1G**	-8.58

Supplementary Fig. 6. Expression levels of MT1G in primary lung cancer tissues and tumorsphere cells. (a) Quantitative RT-PCR analysis of *MT1G* in paired normal and cancerous tissues obtained from NSCLC patients. *MT1G* level in normal tissues was normalized as 1; n=9 paired tissues. **(b,c)** Transcriptome analyses showing downregulated gene expression in lung TICs (TS) compared with non-TICs (N166+). *MT1G* ranked among the top 50 of the 3,300 downregulated genes (b, c). TS, tumorsphere; N166+, CD166+ primary normal lung cells. n=3. **(d)** Luciferase activity of wild-type (UTR wt) or mutant (UTR mut) *MT1G* 3' reporter assay in tumorsphere cells transfected with zip1246, zip1290 or zipCtrl. n=3 replicates. All error bars represent ±SEM and statistical significance was calculated using Student's *t*-test; *p<0.05, **p<0.01; NS, not significant.

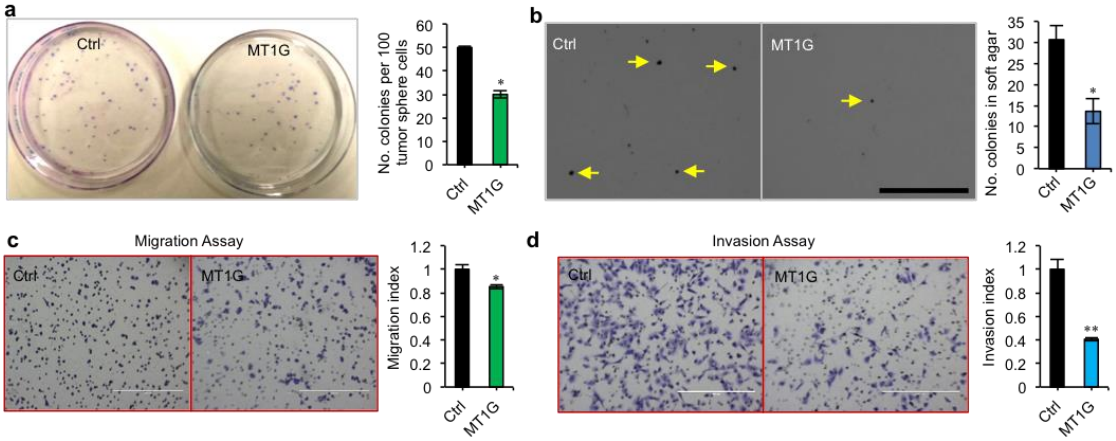

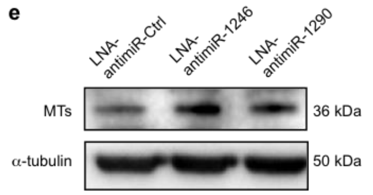

f

Category		Low MTs	High MTs	*p*-value
N	N0	43	61	0.025
	N1-3	21	11	
M	M0	60	72	0.047
	M1	4	0	

Supplementary Fig. 7. MT1G suppresses the colony-forming and invasive abilities of NSCLC *in vitro*. (a) Colony formation assay in adherent conditions of tumorsphere cells overexpressing MT1G or control vector. 100 cells were plated. Colonies were stained with Giemsa (left panel) and quantified (right panel) on day 9; n=3 replicates. **(b)** Soft agar colony formation of tumorsphere cells overexpressing MT1G or control vector. 400 cells were seeded. Colonies as indicated (→) were stained with INT (left panel) and quantified (right panel) on day 28; n=3 replicates. Scale bar, 4 mm. **(c)** Transwell migration assay of tumorsphere cells overexpressing MT1G or control vector. 100,000 cells were seeded. The migrated cells on the lower membrane of Inserts were stained with Giemsa (left panel) and counted (right panel) 6 hours after plating; n=3 replicates. Scale bar, 400 µm. **(d)** Transwell matrigel invasion assay of tumorsphere cells overexpressing MT1G or control vector. 40,000 cells were seeded. The invaded cells on the lower membrane of transwell inserts were stained with Giemsa (left panel) and counted (right panel) 30 hours after plating; n=3 replicates. Scale bar, 400 µm. **(e)** Western blot showing the expression of metallothionein (MTs) protein in subcutaneous xenograft tumors obtained from mice (in Fig. 5f) that were treated with LNA-antimiR-1246, LNA-antimiR-1290 or control. α-tubulin was loaded as endogenous control. **(f)** The association between the intensity of MTs expression by IHC and status of regional lymph nodes (N) or distant metastasis (M) on a NSCLC tissue microarray. n=136. All error bars represent ±SEM and statistical significance was calculated using Student's *t*-test (a,b,c,d) or Chi-squared test (f); **p*<0.05, ***p*<0.01.

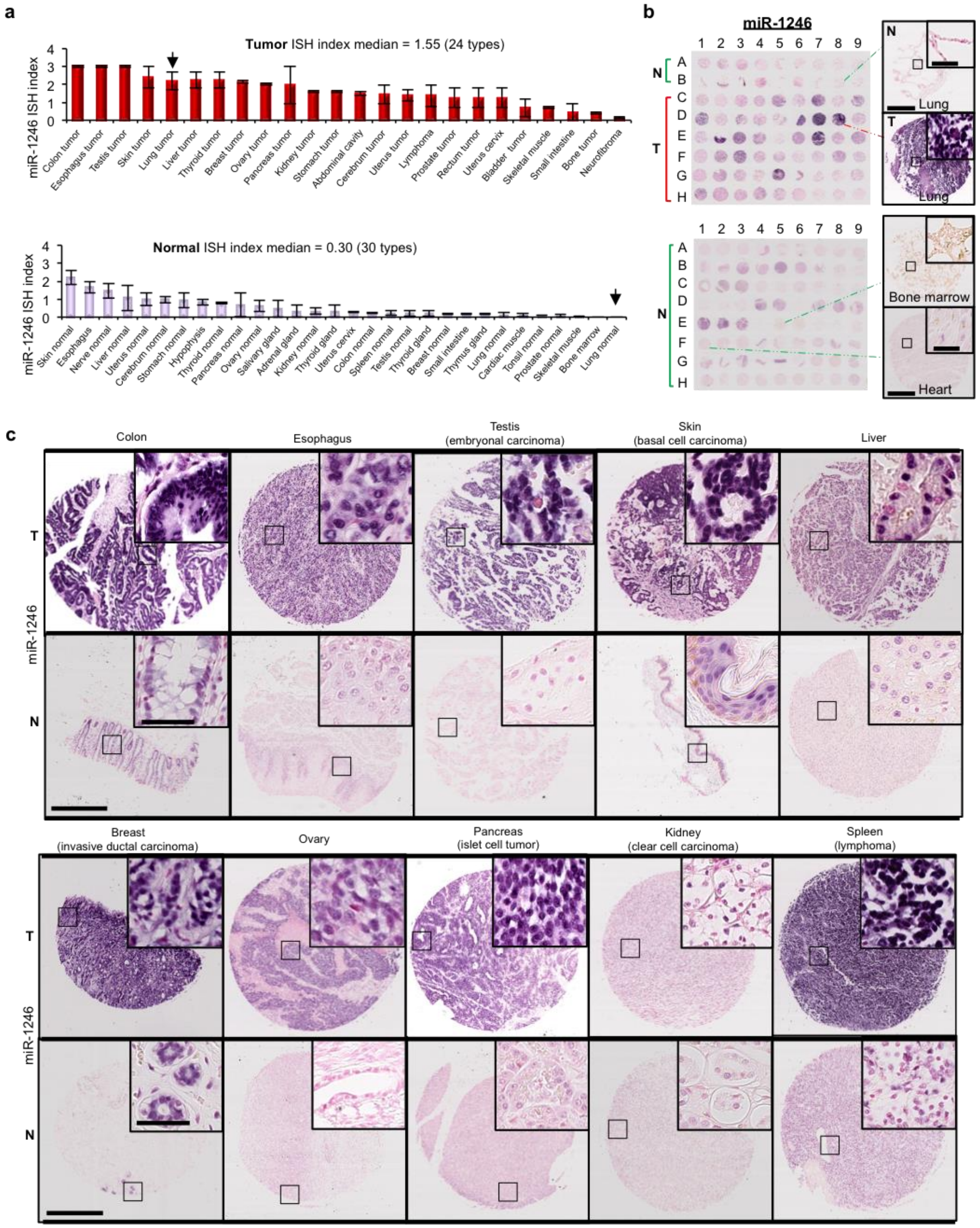

Supplementary Fig. 8. Enriched expression of miR-1246 and miR-1290 are found in a wide variety of tumor types relative to their normal tissue counterparts. (a) ISH index of miR-1246 expression level across different human tumor types (n=24, top panel) and normal tissues (n=30, bottom panel) on a tissue microarray (TMA). ISH index = expression intensity × expression as percentage. All error bars represent +SEM; n=3 replicates. ↓, lung. **(b)** ISH staining for miR-1246 in human tumors and normal tissues in two different TMAs. The candidate images showing miR-1246 expression in low (left panel) and high magnifications (right panel). N, normal. T, tumor. Scale bar, 600 µm (50 µm, inset). **(c)** ISH staining for miR-1246 in paired tumor and normal tissue samples from colon, esophagus, testis, skin and liver (top panels), and from breast, ovary, pancreas, kidney and spleen (bottom panels). Scale bar, 600 µm (50 µm, inset).

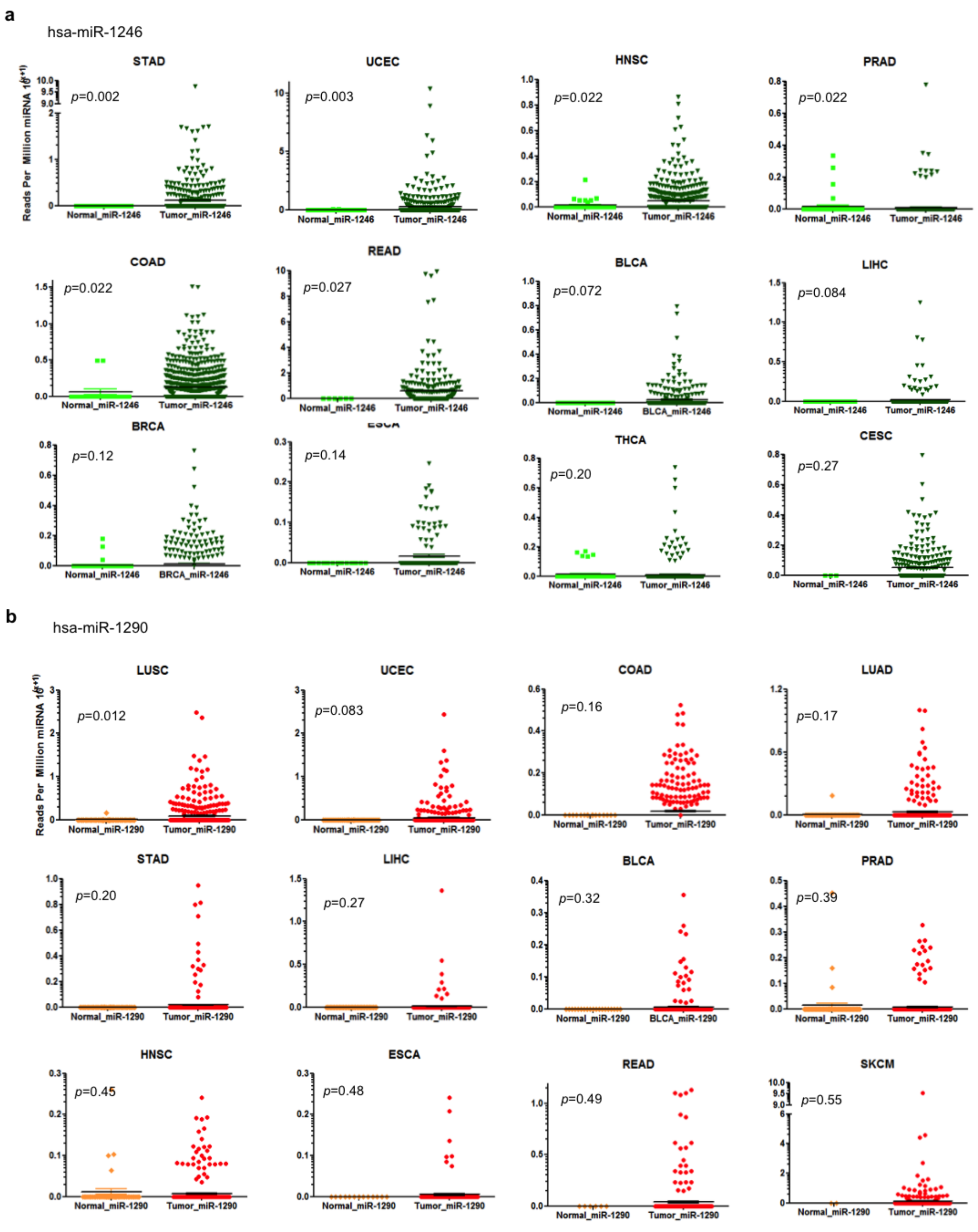

Supplementary Fig. 9. Expression levels of miR-1246 and miR-1290 across various human tumor types based on TCGA miRNA-Seq data. The plots showing the levels of miR-1246 (a) and miR-1290 (b) among up to 12 types of cancers relative to their normal tissues. *p*-values were calculated using Student's *t*-test.

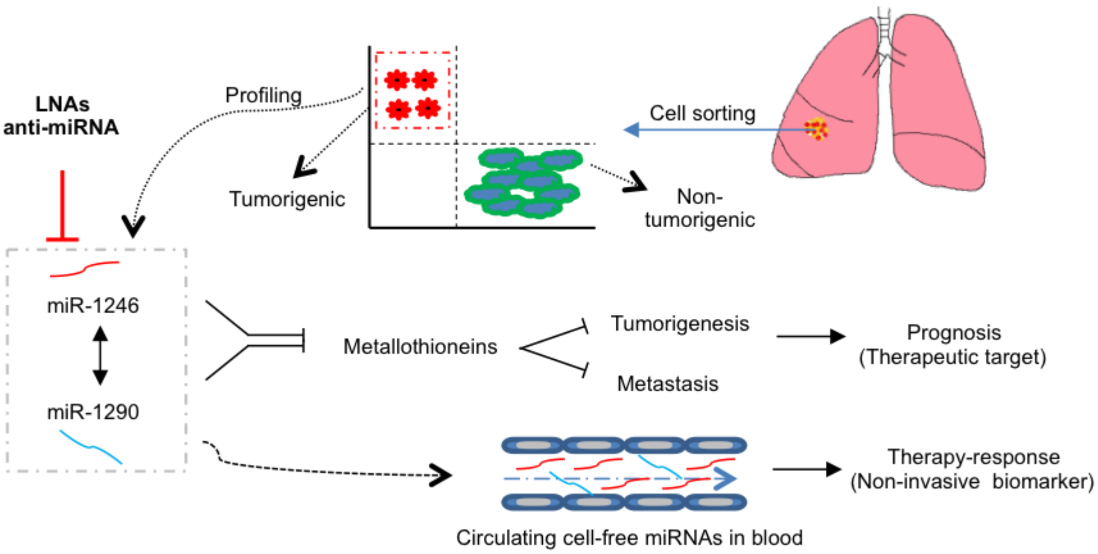

Supplementary Fig. 10. Schematic representation of the roles of miR-1246, miR-1290 and metallothioneins in NSCLC.

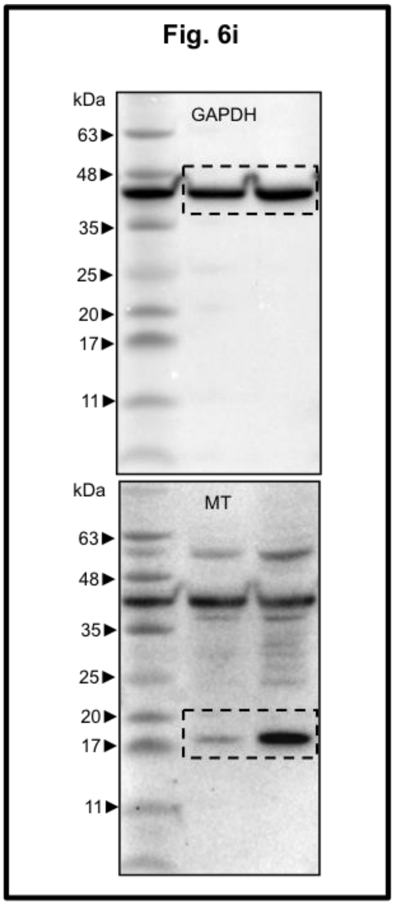

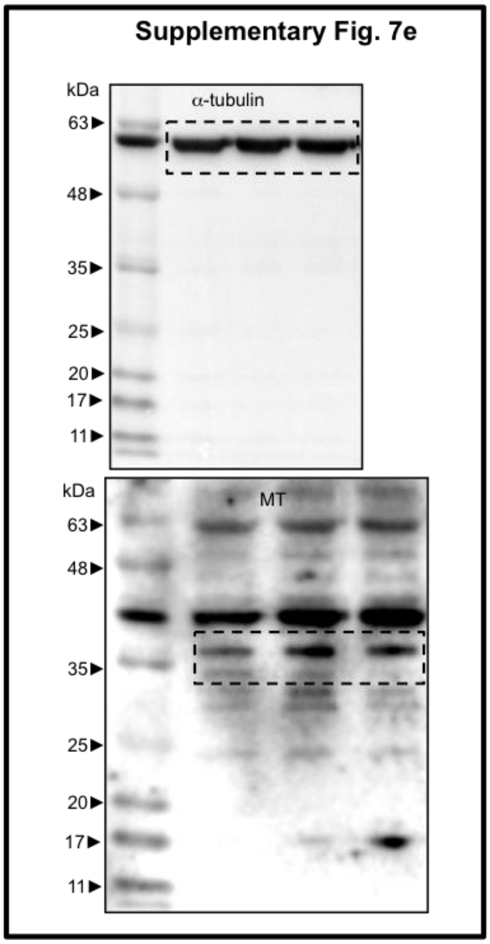

Supplementary Figure 11. Uncropped Western Blots, corresponding to the indicated figures in the manuscript.

Supplementary Tables

Supplementary Table 1. Top-most upregulated and downregulated miRNAs in tumor-initiating cells in non-small cell lung cancer

miRNA	log_2 (TS/(NHBE/SAEC)) (FC, cut-off >2 or <-2)	log_2 (T166+/T166-) (FC, cut-off >1.5 or <-1.5)	Difference
miR-1290	4.5	0.7	up (n=19)
miR-130b	3.6	1.0	
miR-1246	3.6	2.4	
miR-630	3.1	1.1	
miR-196a	2.8	0.7	
miR-196b	2.6	0.7	
miR-9*	2.4	0.7	
miR-106b	2.4	1.4	
miR-93	1.9	1.6	
miR-25	1.9	2.2	
miR-7	1.8	0.9	
miR-18a	1.7	0.6	
miR-192	1.6	0.6	
miR-320c	1.4	0.6	
miR-19a	1.1	0.6	
miR-3667-5p	1.1	0.7	
miR-483-5p	1.0	0.6	
miR-374a	1.0	0.7	
miR-96	1.0	0.6	
miR-377	-1.1	-0.9	down (n=14)
miR-29a	-1.3	-0.6	
miR-376a	-1.4	-0.9	
miR-1260	-1.4	-1.0	
miR-99a	-1.5	-0.7	
miR-1274a	-1.6	-0.6	
miR-125b	-1.8	-1.3	
miR-513a-5p	-1.8	-0.6	
let-7d	-2.1	-1.1	
let-7c	-2.4	-1.7	
let-7i	-2.4	-1.1	
let-7b	-2.7	-1.2	
miR-130a	-3.3	-1.6	
miR-23a	-3.8	-1.0	

Note: TS, tumor sphere; FC, fold change; T, lung cancer patient-derived tumor cells; 166+, CD166+; 166-, CD166-.

Supplementary Table 2. Expression of miR-1246 and miR-1290 across different human cancers based on TCGA miRNA-Seq data

Cancer	Description	miR-1246 (N/T)		miR-1290 (N/T)		No. patients
		FC	*p*-value	FC	*p*-value	
LUSC	Lung squamous cell carcinoma	0.574496	2.71E-08	0.080643	0.012091	504
STAD	Stomach adenocarcinoma	0.114311	0.00196	0.016124	0.198385	443
UCEC	Uterine corpus endometrial Carcinoma	0.250787	0.002741	0.041955	0.083053	548
LUAD	Lung adenocarcinoma	0.107022	0.004347	0.022732	0.171509	521
HNSC	Head and neck squamous cell carcinoma	0.15125	0.021645	-0.01652	0.453623	528
PRAD	Prostate adenocarcinoma	-0.0091	0.021979	-0.00662	0.391253	498
COAD	Colon adenocarcinoma	0.343819	0.024155	0.053833	0.163521	461
READ	Rectum adenocarcinoma	0.559734	0.026974	0.038959	0.490673	171
BLCA	Bladder urothelial carcinoma	0.08001	0.071999	0.017168	0.319218	412
LIHC	Liver hepatocellular carcinoma	0.018394	0.083837	0.009056	0.265112	377
BRCA	Breast invasive carcinoma	0.030557	0.121647	0.001774	0.641866	1098
ESCA	Esophageal carcinoma	0.044802	0.135981	0.014069	0.483665	185
THCA	Thyroid carcinoma	-0.00101	0.203217	-0.01344	1.61E-05	507
CESC	Cervical squamous cell carcinoma and endocervical adenocarcinoma	0.164581	0.26667	0.025542	0.613878	308
SKCM	Skin cutaneous melanoma	0.561717	0.347114	0.11688	0.546408	470
KIRC	Kidney renal clear cell carcinoma	0.006704	0.477279	0.001932	0.617102	536
KICH	Kidney chromophobe	0.003051	0.554628	0	n.a.	66
THYM	Thymoma	0.046651	0.629116	0.022467	0.729072	124
CHOL	Cholangiocarcinoma	0.004948	0.656721	0	NA	36
PAAD	Pancreatic adenocarcinoma	0.011378	0.676258	0.003797	0.852846	185
PCPG	Pheochromocytoma and paraganglioma	0.002318	0.843321	0	n.a.	179

Note: FC, fold change; NA, not applicable; N/T, normal versus tumor. TCGA, The Cancer Genome Atlas.

Supplementary Table 3. Clinical pathologic characteristics of 143 study subjects based on the expression intensity of miR-1246

Expression of miR-1246	Total (n = 143)	Low (n = 30)	High (n = 113)	*p*-value
Mean age (yrs)	63.6 (10.0)	65.5 (7.6)	63.1 (10.5)	0.249
Gender (%)				
Male	97 (68)	23 (77)	74 (65)	0.279
Female	46 (32)	7 (23)	39 (35)	
Histology (%)				
Adenocarcinoma	97 (68)	11 (37)	87 (76)	0.001
Squamous cell carcinoma	46 (32)	19 (63)	27 (24)	
Mean tumor size (cm)	3.79 (2.02)	4.29 (2.22)	3.66 (1.95)	0.141
T stage (%)				
T1	33 (24)	9 (32)	24 (22)	
T2	71 (52)	18 (64)	53 (49)	0.031
T3	16 (12)	0 (0)	16 (15)	
T4	16 (12)	1 (4)	15 (14)	
N stage (%)				
N0	104 (76)	27 (93)	77 (72)	0.024
N1 -3	32 (24)	2 (7)	30 (28)	
M stage (%)				
M0	132 (97)	29 (100)	103 (96)	0.578
M1	4 (3)	0 (0)	4 (4)	
AJCC stage (%)				
I	81 (61)	27 (96)	54 (51)	
II	24 (18)	0 (0)	24 (23)	< 0.001
III	23 (17)	1 (4)	22 (21)	
IV	5 (4)	0 (0)	5 (5)	
Grade (%)				
Well-differentiated	9 (6)	3 (10)	6 (5)	
Moderately differentiated	74 (53)	13 (43)	61 (55)	0. 522
Poorly differentiated	46 (33)	12 (40)	34 (31)	
Undifferentiated	11 (8)	2 (7)	9 (8)	
Status (%)				
Alive	66 (46)	18 (60)	48 (42)	
Lung cancer related death	63 (44)	7 (23)	56 (50)	0.022
Non-lung cancer related death	14 (10)	5 (17)	9 (8)	

Supplementary Table 4. Clinical pathologic characteristics of 143 study subjects based on the expression intensity of miR-1290

Expression of miR-1290	Low (n = 33)	High (n = 110)	*p*-value
Mean age (yrs)	64.6 (7.7)	63.3 (10.6)	0.523
Gender (%)			
Male	23 (70)	74 (67)	0.835
Female	10 (30)	36 (33)	
Histology (%)			
Adenocarcinoma	17 (52)	80 (73)	0.033
Squamous cell carcinoma	16 (48)	30 (27)	
Mean tumor size (cm)	3.27 (1.73)	3.95 (2.08)	0.101
T stage (%)			
T1	12 (39)	21 (20)	
T2	19 (61)	52 (50)	0.001
T3	0 (0)	16 (15)	
T4	0 (0)	16 (15)	
N stage (%)			
N0	31 (97)	73 (70)	0.001
N1 -3	1 (3)	31 (30)	
M stage (%)			
M0	32 (100)	100 (96)	0.573
M1	0 (0)	4 (4)	
AJCC stage (%)			
I	31 (100)	50 (49)	
II	0 (0)	24 (24)	< 0.001
III	0 (0)	23 (23)	
IV	0 (0)	5 (5)	
Grade (%)			
Well-differentiated	4 (12)	5 (5)	
Moderately differentiated	16 (48)	58 (54)	0. 453
Poorly differentiated	10 (30)	36 (34)	
Undifferentiated	3 (9)	8 (7)	
Status (%)			
Alive	17 (52)	49 (45)	
Lung cancer related death	12 (36)	51 (46)	0.567
Non-lung cancer related death	4 (12)	10 (9)	

Supplementary Table 5. Association between CD166 expression and miR-1246 or miR-1290 expression

	CD166 (0~2+)	CD166 (3+)	*p*-value
miR-1246 (0~2+)	154	19	0.004
miR-1246 (3+)	14	8	
miR-1290 (0~2+)	123	18	0.022
miR-1290 (3+)	39	15	

Supplementary Table 6. Tumor response or progression to clinical therapy in NSCLC patients

SN	Clinical assessment of patient status by CT scan (response or progression)	Clinical therapy	Patient ID
1	Response followed by progression (increase in tumor burden or detection of metastasis)	Continuous EGFR TKI	2, 8, 120, 124, 125, 137
2	Progression (increase in tumor burden or detection of metastasis)	Continuous EGFR TKI	1, 105, 215
3	Stable disease (no significant change in tumor burden and no detectable metastasis)	Continuous EGFR TKI	220
4	Progression followed by response (decrease in tumor burden or loss of metastasis)	EGFR TKI, followed by chemotherapy or radiotherapy	122, 128, 134, 218, 219

Note: CT, computed tomography; EGFR, epidermal growth factor receptor; TKI, tyrosine kinase inhibitor.

Supplementary Table 7. Clinical pathologic characteristics of 143 study subjects based on the expression intensity of MT

Expression of MT	Low (n = 67)	High (n = 76)	*p*-value
Mean age (yrs)	61.4 (10.9)	65.5 (8.7)	0.014
Gender (%)			
Male	43 (64)	54 (71)	0.473
Female	24 (36)	22 (29)	
Histology (%)			
Adenocarcinoma	53 (79)	44 (58)	0.007
SCC	14 (21)	32 (42)	
Mean tumour size (cm)	3.87 (2.18)	3.73 (1.88)	0.684
T stage (%)			
T1	11 (17)	22 (31)	
T2	28 (44)	43 (60)	0.001
T3	13 (20)	3 (4)	
T4	12 (19)	4 (6)	
N stage (%)			
N0	43 (67)	61 (85)	0.025
N1 -3	21 (33)	11 (15)	
M stage (%)			
M0	60 (94)	72 (100)	0.047
M1	4 (6)	0 (0)	
AJCC stage (%)			
I	25 (40)	56 (79)	
II	18 (29)	6 (8)	< 0.001
III	14 (23)	9 (13)	
IV	5 (8)	0 (0)	
Grade (%)			
Well-differentiated	4 (6)	5 (7)	
Moderately differentiated	38 (58)	36 (48)	0. 059
Poorly differentiated	15 (23)	31 (41)	
Undifferentiated	8 (12)	3 (4)	
Status (%)			
Alive	32 (48)	34 (45)	
Lung cancer related death	29 (43)	34 (45)	0.912
Non-lung cancer related death	6 (9)	8 (10)	

Supplementary Table 8. Expression of miR-1246 and miR-1290 in a tissue microarray FDA808-2

position	organ	Pathology	grade	stage	tnm	type	miR-1246		miR-1290	
							Int. (+)	Pct. (%)	Int. (+)	Pct. (%)
A1	Uterus	Normal endometrium tissue	-	-	-	N	2	75	1	70
A2	Uterus	Normal endometrium tissue	-	-	-	N	3	90	3	90
A3	Uterus	Normal endometrium tissue	-	-	-	N	2	90	2	100
A4	Uterine cervix	Cancer adjacent normal cervical canals tissue	-	-	-	NAT	0	0	0	0
A5	Uterine cervix	Normal cervical canals tissue	-	-	-	N	0	0	1	20
A6	Uterus	Cancer adjacent normal cervix tissue	-	-	-	NAT	1	5	0	0
A7	Skeletal muscle	Normal skeletal muscle tissue	-	-	-	N	1	80	0	0
A8	Skeletal muscle	Normal skeletal muscle tissue	-	-	-	N	0	0	0	0
A9	Thyroid	Normal thyroid gland tissue (with hyperplasia in follicles)	-	-	-	N	1	80	0	0
B1	Skin	Normal skin tissue	-	-	-	N	2	90	0	0
B2	Skin	Normal skin tissue	-	-	-	N	3	100	0	0
B3	Skin	Normal skin tissue	-	-	-	N	2	90	2	80
B4	Nerve	Normal peripheral nerve tissue	-	-	-	N	2	80	2	5
B5	Nerve	Normal peripheral nerve tissue	-	-	-	N	1	70	0	0
B6	Nerve	Normal peripheral nerve tissue	-	-	-	N	3	70	0	0
B7	Lung	Normal lung tissue (pneumonia)	-	-	-	N	0	0	0	0
B8	Lung	Normal lung tissue	-	-	-	N	0	0	0	0
B9	Lung	Normal lung tissue	-	-	-	N	2	20	1	5
C1	Cerebrum	Glioblastoma	-	-	-	T	2	80	0	0
C2	Cerebrum	Atypical meningioma	-	-	-	T	1	70	1	75
C3	Cerebrum	Malignant ependymoma	-	-	-	T	1	80	2	80
C4	Cerebrum	Malignant oligodendroglioma	-	-	-	T	3	90	3	70
C5	Ovary	Serous adenocarcinoma	3	II	T2N0M0	T	2	100	2	100
C6	Ovary	Adenocarcinoma	3	III	T3N0M0	T	2	100	2	90

C7	Pancreas	Islet cell carcinoma	-			T	3	100	3	100
C8	Pancreas	Adenocarcinoma	3	II	T2N0M0	T	1	90	2	90
C9	Testis	Seminoma	-	I	T1N0M0	T	3	100	3	100
D1	Testis	Embryonal carcinoma	-	I	T2N0M0	T	3	100	1	65
D2	Thyroid	Medullary carcinoma	-	II	T3N0M0	T	2	90	n.a.	n.a.
D3	Thyroid	Papillary carcinoma	-	III	T2aN1M0	T	3	90	2	10
D4	Breast	Intraductal carcinoma with early infiltrate	-	0	TisN0M0	T	2	80	2	100
D5	Breast	Invasive ductal carcinoma	2	IIa	T2N0M0	T	2	90	3	90
D6	Breast	Invasive ductal carcinoma	2	IIb	T2N1M0	T	3	100	3	100
D7	Spleen	Diffuse B-cell lymphoma	-	-	-	T	3	100	3	100
D8	Lung	Small cell undifferentiated carcinoma	-	I	T2N0M0	T	3	100	3	100
D9	Lung	Squamous cell carcinoma	3	I	T2N0M0	T	2	90	2	90
E1	Lung	Adenocarcinoma	2	II	T2N1M0	T	2	90	2	15
E2	Esophagus	Squamous cell carcinoma	2	IIa	T2N0M0	T	3	100	2	70
E3	Esophagus	Adenocarcinoma	3	IIa	T3N0M0	T	3	100	2	90
E4	Stomach	Mucinous adenocarcinoma	3	II	T2N1M0	T	2	80	3	90
E5	Small intestine	Adenocarcinoma	2	II	T3N0M0	T	0	0	n.a.	n.a.
E6	Small intestine	Malignant interstitialoma	-	IIb	T2N0M0	T	1	90	0	0
E7	Colon	Adenocarcinoma	2	II	T4N0M0	T	3	100	3	100
E8	Abdominal cavity	Interstitialoma	-	IIb	T2N0M0	T	2	80	2	80
E9	Rectum	Adenocarcinoma	2	I	T2N0M0	T	2	90	2	100
F1	Rectum	Intermediate grade malignant interstitialoma	-	IIb	T2N0M0 G2	T	1	70	0	0
F2	Liver	Hepatocellular carcinoma	1	III	T3N0M0	T	2	90	2	70
F3	Liver	Hepatoblastoma	-	-	-	T	3	90	3	100
F4	Kidney	Clear cell carcinoma	2	I	T1N0M0	T	2	80	2	80
F5	Prostate	Adenocarcinoma	3	II	T2N0M0	T	2	90	0	0
F6	Prostate	Adenocarcinoma	2	II	T2N0M0 G4	T	1	70	1	80
F7	Uterus	Leiomyoma	-	IIa	T1bN0M0	BT	1	80	1	80
F8	Uterus	Adenocarcinoma endometrium	3	IIa	T2aN0M0	T	2	90	2	100
F9	Uterus	Clear cell carcinoma with necrosis	-	IIb	T2bN0M0	T	2	90	2	90

G1	Uterine cervix	Squamous cell carcinoma	3	IIIb	T1bN1M0	T	1	70	1	40
G2	Uterine cervix	Squamous cell carcinoma	3	II	T2N0M0	T	2	90	2	90
G3	Striated muscle	Embryonal rhabdomyosarcom of left leg	-	Ia	T1aN0M0	T	1	70	1	5
G4	Rectum	Malignant melanoma	-	II	T4N0M0	T	1	60	1	40
G5	Skin	Basal cell carcinoma of head	-	I	T2N0M0	T	3	100	3	90
G6	Skin	Squamous cell carcinoma of left chest wall	2	II	T3N0M0	T	2	90	2	80
G7	Soft tissue	Neurofibroma	-	-	-	BT	1	15	0	0
G8	Retroperi toneum	Neuroblastoma	-	IIIb	T2bN0M0	T	2	10	3	10
G9	Abdomin al cavity	Malignant mesothelioma	-	I	T2N0M0	T	2	70	2	60
H1	Mediasti num	Diffuse B-cell lymphoma	-	-	-	T	2	90	1	50
H2	Lymph node	Diffuse B cell lymphoma of right thigh	-	-	-	T	1	40	1	60
H3	Lymph node	Hodgkin's lymphoma	-	-	-	T	1	50	1	80
H4	Pelvic cavity	Large cell anaplastic lymphoma	-	-	-	T	2	80	2	70
H5	Bladder	Transitional cell carcinoma	3	II	T3aN0M0	T	2	60	2	75
H6	Bladder	Low grade malignant leiomyosarcoma	-	Ib	T2bN0M0 G1	T	1	20	1	60
H7	Bone	Osteosarcoma of right inferior extremity femur	-	IIIb	T2N0M0	T	1	40	2	60
H8	Retroperi toneum	Spindle cell rhabdomyosarcoma	-	IIb	T2N0M0 G2	T	0	0	1	30
H9	Smooth muscle	Intermediate grade malignant leiomyosarcoma of left buttock	-	IIIb	T2bN0M0	T	0	0	1	40

Note: N, normal; T, malignant tumor; NAT, cancer adjacent normal; n.a., not applicable; BT, benign tumor; Grade (1, well-differentiated; 2, moderately-differentiated; 3, poorly differentiated; 4, undifferentiated); TNM grading (T, primary tumor; N, regional lymph nodes; M, distant metastasis).

Supplementary Table 9. Expression of miR-1246 and miR-1290 in a tissue microarray FDA808-1

position	organ	Pathology	type	miR-1246		miR-1290	
				Int. (+)	Pct. (%)	Int. (+)	Pct. (%)
A1	Cerebrum	Normal cerebrum tissue	N	1	50	0	0
A2	Cerebrum	Normal cerebrum tissue	N	2	90	0	0
A3	Cerebrum	Normal cerebrum tissue	N	3	20	0	0
A4	Cerebellum	Normal cerebellum tissue	N	1	100	0	0
A5	Cerebellum	Normal cerebellum tissue	N	1	100	0	0
A6	Cerebellum	Normal cerebellum tissue	N	1	100	0	0
A7	Adrenal gland	Normal adrenal gland tissue	N	0	0	0	0
A8	Adrenal gland	Normal adrenal gland tissue	N	0	0	0	0
A9	Adrenal gland	Normal adrenal gland tissue	N	1	100	2	75
B1	Ovary	Normal ovary tissue	N	1	100	0	0
B2	Ovary	Normal ovary tissue	N	0	0	0	0
B3	Ovary	Normal ovary tissue	N	1	90	0	0
B4	Pancreas	Normal pancreas tissue	N	1	5	1	80
B5	Pancreas	Normal pancreas tissue	N	2	100	0	0
B6	Pancreas	Normal pancreas tissue	N	0	0	0	0
B7	Parathyroid gland	Normal thyroid gland tissue	N	1	100	3	80
B8	Parathyroid gland	Normal thyroid gland tissue	N	0	0	2	70
B9	Parathyroid gland	Normal parathyroid gland tissue	N	0	0	1	30
C1	Hypophysis	Normal hypophysis tissue	N	1	100	0	0
C2	Hypophysis	Normal hypophysis tissue	N	1	100	0	0
C3	Hypophysis	Normal hypophysis tissue	N	1	50	0	0
C4	Testis	Normal testis tissue	N	1	50	0	0
C5	Testis	Normal testis tissue	N	0		0	0
C6	Testis	Normal testis tissue	N	1	10	0	0
C7	Thyroid gland	Normal thyroid gland tissue	N	1	50	2	40
C8	Thyroid gland	Normal thyroid gland tissue	N	1	10	2	50
C9	Thyroid gland	Normal thyroid gland tissue	N	0		2	10
D1	Breast	Normal breast tissue	N	1	10	0	0
D2	Breast	Normal breast tissue	N	2	10	0	0
D3	Breast	Normal breast tissue	N	2	10	0	0
D4	Spleen	Normal spleen tissue	N	2	10	0	0
D5	Spleen	Normal spleen tissue	N	1	50	0	0
D6	Spleen	Normal spleen tissue	N	0	0	2	70
D7	Tonsil	Normal tonsil tissue	N	1	10	2	70
D8	Tonsil	Normal tonsil tissue	N	1	10	2	80
D9	Tonsil	Normal tonsil tissue	N	1	10	1;2	60
E1	Thymus gland	Normal thymus gland tissue	N	1	20	0	0
E2	Thymus gland	Normal thymus gland tissue	N	1	20	0	0
E3	Thymus gland	Normal thymus gland tissue	N	1	10	0	0
E4	Bone marrow	Normal myeloid tissue	N	0	0	0	0
E5	Bone marrow	Normal myeloid tissue	N	0	0	0	0

E6	Bone marrow	Normal myeloid tissue	N	0	0	0	0
E7	Lung	Normal lung tissue	N	0	0	2	10
E8	Lung	Normal lung tissue	N	0	0	3	40
E9	Lung	Normal lung tissue	N	0	0	0	0
F1	Heart	Normal cardiac muscle tissue	N	1	30	0	0
F2	Heart	Normal cardiac muscle tissue	N	0	0	0	0
F3	Heart	Normal cardiac muscle tissue	N	1	5	0	0
F4	Esophagus	Normal esophagus tissue	N	2	100	0	0
F5	Esophagus	Normal esophagus tissue	N	2	100	1	20
F6	Esophagus	Normal esophagus tissue	N	2	50	1	30
F7	Stomach	Normal stomach tissue	N	2	50	2	20
F8	Stomach	Normal stomach tissue	N	2	80	3	40
F9	Stomach	Normal stomach tissue (smooth muscle tissue)	N	1	20	2	30
G1	Small intestine	Normal small intestine tissue	N	1	10	1	60
G2	Small intestine	Normal small intestine tissue	N	2	10	1	60
G3	Small intestine	Normal small intestine tissue	N	2	10	2	75
G4	Colon	Normal colon tissue	N	2	10	0	0
G5	Colon	Normal colon tissue	N	3	10	2	10
G6	Colon	Normal colon tissue	N	2	10	1	25
G7	Liver	Normal liver tissue	N	1	80	1	80
G8	Liver	Normal liver tissue	N	0	0	2	75
G9	Liver	Normal liver tissue	N	3	80	3	90
H1	Salivary gland	Normal salivary gland tissue	N	2	70	0	0
H2	Salivary gland	Normal salivary gland tissue	N	0	0	1	15
H3	Salivary gland	Normal salivary gland tissue	N	0	0	0	0
H4	Kidney	Normal kidney tissue	N	0	0	1	10
H5	Kidney	Normal kidney tissue	N	1	50	2	15
H6	Kidney	Normal kidney tissue	N	1	50	1	10
H7	Prostate	Normal prostate tissue	N	0	0	0	0
H8	Prostate	Normal prostate tissue	N	0	0	2	90
H9	Prostate	Normal prostate tissue	N	2	10	2	90

Note: N, normal; Int., intensity; Pct., percentage; n.a., not applicable.

Supplementary Table 10. Expression of miR-1246 and miR-1290 in eight types of cancers by ISH in a tissue microarray BC00112

position	organ	Pathology	grade	type	miR-1246		miR-1290	
					Int. (+)	Pct. (%)	Int. (+)	Pct. (%)
A1	Liver	Hepatocellular carcinoma	I	T	3	90	2	90
A2	Colon	Adenocarcinoma	I	T	3	90	2	70
A3	Stomach	Adenocarcinoma	II	T	2	60	0	0
A4	Esophagus	Squamous cell carcinoma	I	T	3	70	2	20
A5	Intestine	Adenocarcinoma	I	T	2	90	0	0
A6	Pancreas	Cancer adjacent tissue	-	N	0	0	0	0
A7	Lung	Squamous cell carcinoma	II	T	1	20	0	0
A8	Epiploon	Metastatic mucinous adenocarcinoma	II	Mets	0	0	0	0
B1	Liver	Hepatocellular carcinoma	II	T	2	90	0	0
B2	Colon	Adenocarcinoma	I-II	T	1	90	0	0
B3	Stomach	Adenocarcinoma	III	T	2		1	20
B4	Esophagus	Squamous cell carcinoma	II	T	3	90	2	20
B5	Intestine	Adenocarcinoma	II	T	1	90	0	0
B6	Pancreas	Duct adenocarcinoma	II	T	2	80	0	0
B7	Lung	Squamous cell carcinoma	III	T	3	80	1	10
B8	Epiploon	Metastatic adenocarcinoma	III	Mets	n.a.	n.a.	n.a.	n.a.
C1	Liver	Hepatocellular carcinoma	II	T	n.a.	n.a.	n.a.	n.a.
C2	Colon	Mucinous adenocarcinoma	III	T	0	0	0	0
C3	Stomach	Adenocarcinoma	III	T	3	50	0	0
C4	Esophagus	Squamous cell carcinoma	II	T	2	50	1	80
C5	Intestine	Adenocarcinoma	III	T	1	40	0	0
C6	Pancreas	Islet cell carcinoma	-	T	2	20	0	0
C7	Lung	Atypical carcinoid	-	T	2	20	1	5
C8	Epiploon	Metastatic adenocarcinoma	III	Mets	0	0	0	0
D1	Liver	Cancer adjacent normal hepatic tissue	-	N	2	80	0	0
D2	Colon	Cancer adjacent normal colonic tissue	-	N	3	30	0	0
D3	Stomach	Cancer adjacent normal gastric tissue	-	N	1	10	0	0
D4	Esophagus	Cancer adjacent normal esophageal tissue	-	N	0	0	0	0
D5	Intestine	Cancer adjacent normal small intestinal tissue	-	N	2	90	0	0
D6	Pancreas	Cancer adjacent normal pancreatic tissue	-	N	1	30	0	0
D7	Lung	Cancer adjacent normal lung tissue	-	N	0	0	0	0
D8	Epiploon	Cancer adjacent normal greater omentum tissue	-	N	0	0	0	0

E1	Liver	Cancer adjacent normal hepatic tissue	-	N	1	90	0	0
E2	Colon	Cancer adjacent normal colonic tissue	-	N	2	90	0	0
E3	Stomach	Cancer adjacent normal gastric tissue	-	N	0	0	0	0
E4	Esophagus	Cancer adjacent normal esophageal tissue	-	N	2	80	0	0
E5	Intestine	Cancer adjacent normal small intestinal tissue	-	N	n.a.	n.a.	n.a.	n.a.
E6	Pancreas	Cancer adjacent normal pancreatic tissue	-	N	1	10	0	0
E7	Lung	Cancer adjacent normal lung tissue	-	N	0	0	0	0
E8	Epiploon	Cancer adjacent normal greater omentum tissue	-	N	0	0	0	0
F1	Liver	Cancer adjacent normal hepatic tissue	-	N	2	80	0	0
F2	Colon	Cancer adjacent colonic mucosa tissue (chronic inflammation)	-	N	1	80	0	0
F3	Stomach	Cancer adjacent normal gastric tissue	-	N	0	0	0	0
F4	Esophagus	Cancer adjacent normal esophageal tissue	-	N	3	80	2	70
F5	Intestine	Cancer adjacent normal small intestinal tissue	-	N	1	70	0	0
F6	Pancreas	Cancer adjacent normal pancreatic tissue	-	N	0	0	0	0
F7	Lung	Cancer adjacent normal lung tissue	-	N	0	0	0	0
F8	Epiploon	Cancer adjacent normal greater omentum tissue	-	N	0	0	0	0

Note: N, normal; T, malignant tumor; Mets, metastasis; Int., intensity; Pct., percentage; n.a., not applicable.
